# Diosgenin restores memory function via SPARC-driven axonal growth from the hippocampus to the PFC in Alzheimer’s disease model mice

**DOI:** 10.1038/s41380-023-02052-9

**Published:** 2023-04-22

**Authors:** Ximeng Yang, Chihiro Tohda

**Affiliations:** Section of Neuromedical Science, Institute of Natural Medicine, University of Toyama, 2630 Sugitani, Toyama, 930-0194 Japan

**Keywords:** Neuroscience, Psychiatric disorders, Molecular biology

## Abstract

Central nervous system axons have minimal capacity to regenerate in adult brains, hindering memory recovery in Alzheimer’s disease (AD). Although recent studies have shown that damaged axons sprouted in adult and AD mouse brains, long-distance axonal re-innervation to their targets has not been achieved. We selectively visualized axon-growing neurons in the neural circuit for memory formation, from the hippocampus to the prefrontal cortex, and showed that damaged axons successfully extended to their native projecting area in mouse models of AD (5XFAD) by administration of an axonal regenerative agent, diosgenin. In vivo transcriptome analysis detected the expression profile of axon-growing neurons directly isolated from the hippocampus of 5XFAD mice. Secreted protein acidic and rich in cysteine (SPARC) was the most expressed gene in axon-growing neurons. Neuron-specific overexpression of SPARC via adeno-associated virus serotype 9 delivery in the hippocampus recovered memory deficits and axonal projection to the prefrontal cortex in 5XFAD mice. DREADDs (Designer receptors exclusively activated by designer drugs) analyses revealed that SPARC overexpression-induced axonal growth in the 5XFAD mouse brain directly contributes to memory recovery. Elevated levels of SPARC on axonal membranes interact with extracellular rail-like collagen type I to promote axonal remodeling along their original tracings in primary cultured hippocampal neurons. These findings suggest that SPARC-driven axonal growth in the brain may be a promising therapeutic strategy for AD and other neurodegenerative diseases.

## Introduction

Alzheimer’s disease (AD) is a progressing neurodegenerative dementia characterized by the deposition of amyloid β (Aβ) and neural disruption in the brain [1]. The worldwide incidence of sporadic AD is estimated to reach 150 million by 2050, becoming a refractory problem. Although anti-AD candidate drugs have been developed targeting Aβ reduction, most clinical studies have failed to recover memory function [2, 3], suggesting the limitations of this strategy. Therefore, we hypothesized that repairing damaged neural networks is indispensable to normalize neural function and recover memory deficits in AD.

Since damaged axons in the central nervous system (CNS) are believed to have minimal regenerative capacity, few groups have attempted to regenerate axons in injured brains [4]. Meanwhile, recent studies have demonstrated that axons spontaneously regrew or sprouted after injury in adult and AD mouse brains [5–7]. However, those studies focused on axonal projection in local microcircuits. It remains unknown whether long-distance axonal re-innervation is possible in the brain.

Therefore, we investigated long-distance axonal growth in the brain in a mouse model of AD, 5XFAD [8]. The 5XFAD mice co-express five familial AD mutants of human amyloid precursor protein and presenilin-1 specifically in neurons, which facilitate pathological progression of AD. As an axonal regenerative agent, we focused on diosgenin, a steroid sapogenin. In our previous studies, diosgenin promoted axonal growth and regeneration in normal and Aβ-treated cultured neurons, and induced memory enhancement in normal mice [9], 5XFAD mice [10], and healthy humans [11]. Importantly, diosgenin administration reduces abnormally swollen axons in 5XFAD mouse brains [10]. Diosgenin also increases cross-correlational spike firing between the hippocampal (HPC) CA1 and the prefrontal cortex (PFC) in normal mice [9]. Pharmacokinetically, diosgenin is delivered to the blood and brain after oral administration [11–13]. Signal pathway of diosgenin is initiated by activation of 1,25D_3_-membrane-associated rapid response steroid-binding receptor (MARRS), and reduction in heat shock cognate 70 in neurons [14, 15]. However, the phenomenon and molecular mechanism of correct pathfinding of axons driven by diosgenin remains unknown.

This study determined that diosgenin administration promotes the extension of long axons from the HPC to the PFC, which is involved in neural circuits for memory formation. Subsequently, molecular mechanisms of accurate pathfinding of extending axons were elucidated. Our study suggests notable therapeutic strategies and clinical adaptations for AD.

## Materials and methods

See Supplementary information for the following methods: Mice, Diosgenin administration, Retrograde labeling using Dextran 3000 MW, LCM and DNA microarray, Primary neuron culture and immunocytochemistry, Western blot, siRNA transfection, AAV9 injection, Behavioral tests, Designer receptors exclusively activated by designer drugs (DREADDs) experiments, Anterograde labeling using BDA, Immunohistochemistry, Collagen type I coating and measurement of axonal length, Live cell imaging using triple chamber neuron device, Image analysis, and Statistical analysis.

## Results

### Diosgenin promotes axonal growth in 5XFAD mouse

We assessed whether diosgenin has axonal growth effect in vivo using cortex-axotomized mice. No neurofilament-H (NF-H)-positive staining was detected in the lesion area at 1 h following axotomy (Supplementary Fig. 1A). To avoid overlaid estimation of neuroprotection and axonal growth, diosgenin administration started from 7 days after axotomy. Since administration of diosgenin for 14–20 days recovered memory deficits in 5XFAD mice [10, 14], diosgenin or a vehicle solution was administered for 15 days. Axonal density in cortical layers I–IV was quantified in the lesion area. Diosgenin administration significantly promoted axonal growth (Supplementary Fig. 1B), with no significant change in the lesion area size (Supplementary Fig. 1C).

**Fig. 1:**
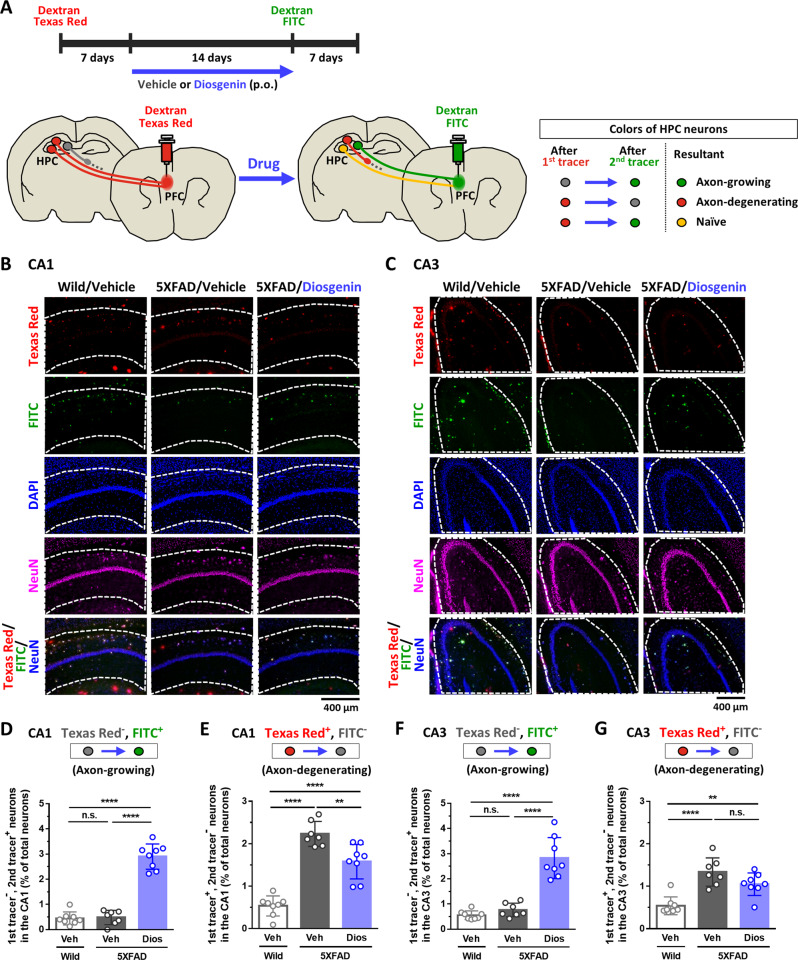
Diosgenin administration promotes axonal growth in 5XFAD mice brain. **A** Seven days after Dextran Texas Red (1st tracing) was injected into the PFC, diosgenin or vehicle solution was orally administered to wild-type and 5XFAD mice once a day for 14 days. Dextran FITC (2nd tracing) was further injected into the PFC at 7 days before sacrifice. Images for Dextran Texas Red, Dextran FITC, DAPI, and NeuN staining in the CA1 (**B**) and CA3 (**C**) were shown. **D**–**G** The number of axon-growing (Texas Red^-^, FITC^+^, NeuN^+^, DAPI^+^) neurons and axon-degenerating (Texas Red^+^, FITC^-^, NeuN^+^, DAPI^+^) neurons in the CA1 and CA3 were quantified. **p < 0.01, ****p < 0.0001, one-way ANOVA *post-hoc* Bonferroni test, mean ± standard deviation, wild-type mice (Wild)/vehicle (Veh), *n* = 8; 5XFAD mice (5XFAD)/Veh, *n* = 7; 5XFAD/diosgenin (Dios), *n* = 8. **D** Effect size (*r*) = 0.959, power (1 − *β*) = 0.975, **E** *r* = 0.918, 1 − *β* = 0.963, **F** *r* = 0.908, 1 − *β* = 0.959, **G** *r* = 0.792, 1 − *β* = 0.893. High magnification images are shown in Supplementary Figs. 3 and 4.

Next, we focused on a long axonal tract related to memory formation, from the HPC to the PFC [16, 17] in 5XFAD mice. Since neural circuit from the dorsal HPC, but not the ventral HPC, to the PFC plays a role in short-term working memory [18], axon-growing neurons were visualized by sequentially injecting two colors of retrograde Dextran tracers into the PFC to determine whether diosgenin administration promotes axonal growth from the dorsal HPC in 5XFAD mouse (Fig. 1A–C). Seven days prior to initiating drug administration, the originally projected axons from the HPC to the PFC were labeled with Dextran Texas Red. After 14-day diosgenin administration, Dextran fluorescein isothiocyanate (FITC), was injected in exactly the same PFC position of the first tracer; thus, naive neurons were labeled with two tracers (merged as yellow), and axon-growing neurons were labeled with Dextran FITC only. Complete match of the injected positions for the two tracers was confirmed for all analyzed mice (Supplementary Fig. 2A). When these tracers were sequentially injected into the PFC, they spread similarly to the HPC (Supplementary Fig. 2B), confirming the accuracy of the injecting techniques and analogous spreading ranges of two tracers. The dentate gyrus was excluded from the analyses to exclude neurogenesis-induced increase of axonal projection [19]. Tracer-positive neuronal cell bodies were determined by neuronal nuclei (NeuN)- and 4’,6-diamidino-2-phenylindole (DAPI)- copositive staining. The number of mono-positive neurons for the second tracer Dextran FITC in the CA1 and CA3 regions were significantly increased by diosgenin administration (Fig. 1D, F), showing that diosgenin promoted axonal growth in 5XFAD mice. Interestingly, axon-degenerating neurons (Texas Red^+^, FITC^-^) was significantly increased in 5XFAD compared with wild-type mice; however, axonal degeneration was significantly lower in the CA1 (Fig. 1E) and tended to be lower in the CA3 (Fig. 1G) by diosgenin administration. The number of naive neurons (Texas Red^+^, FITC^+^) was tended to be increased by diosgenin administration in 5XFAD mice, indicating the axon-protective effect of diosgenin (Supplementary Fig. 3A, 4A). The originally projected axons (total Texas Red^+^) in vehicle- and diosgenin-administered 5XFAD mice did not differ, but it was significantly lower than that of wild-type mice, showing that the same degree of axonal retraction in this neural circuit had occurred in 5XFAD mice before drug administrations (Supplementary Figs. 3B and 4B). The number of total FITC^+^ neurons was significantly decreased in vehicle-treated 5XFAD compared with wild-type mice, whereas it was significantly increased by diosgenin, suggesting that the axonal density in this circuit was recovered to equal level as wild-type in diosgenin-treated 5XFAD mice. The number of total NeuN^+^ neurons and DAPI^+^ cells in the CA1 and CA3 did not differ among the groups (Supplementary Figs. 3D, E and 4D, E). High magnification images of the CA1 and CA3 were shown in Supplementary Figs. 3 and 4.

### In vivo transcriptome to assess gene expression profile of axon-growing neurons

**Fig. 2:**
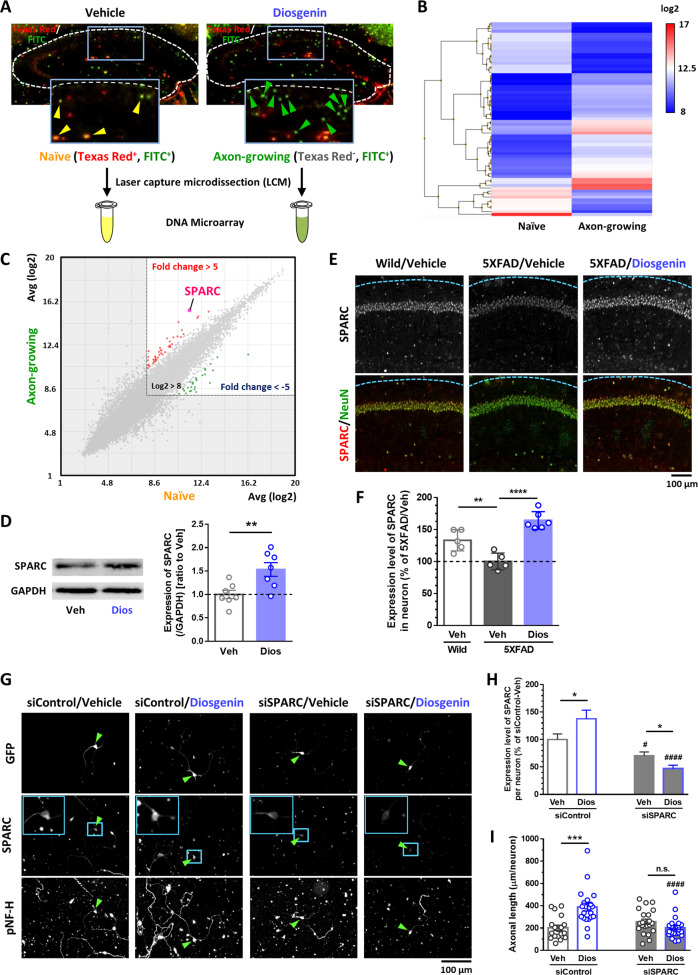
Gene profile of axon-growing neurons by in vivo transcriptome. **A** Naive neurons (Texas Red^+^, FITC^+^) of vehicle-treated 5XFAD mice (*n* = 3) and axon-growing neurons (Texas Red^-^, FITC^+^) of diosgenin-treated 5XFAD mice (*n* = 3) were individually isolated by laser capture microdissection. Total RNA was extracted from each pool of neurons to use for DNA microarray. **B**, **C** Using transcriptome analysis console, we compared the gene expression profiles in Hierarchical clustering (**B**) and Scatter plot (**C**) between naive neurons and axon-growing neurons (log2 > 8, fold change > 5). SPARC was detected as the gene with the highest expression in axon-growing neurons. **D** Mouse primary HPC neurons were cultured for 3 days and then treated with diosgenin (1 µM) or vehicle solution for 4 days. The neuron lysates were used for western blot analysis. The expression level of SPARC (/GAPDH) was quantified for each neuron lysate. **p < 0.01, two-tailed unpaired *t*-test, mean ± standard error, vehicle (Veh), *n* = 7; diosgenin (Veh), *n* = 7 lysates. Effect size (*r*) = 0.670, power (1 − *β*) = 0.873. The image of the complete gel is shown in Supplementary Fig. 5. **E**, **F** Diosgenin or vehicle solution was orally administered to wild-type and 5XFAD mice once a day for 14 days. **F** The expression level of SPARC in CA1 NeuN^+^ neurons was quantified using immunocytochemistry. **p < 0.01, ****p < 0.0001, one-way ANOVA *post-hoc* Bonferroni test, mean ± standard deviation, wild-type mice (Wild)/Veh, *n* = 5; 5XFAD mice (5XFAD)/Veh, *n* = 5; 5XFAD/Dios, *n* = 6. *r* = 0.891, 1 − *β* = 0.820. **G**–**I** siRNA for SPARC (30 nM; siSPARC) or control siRNA (30 nM; siControl) was transfected together with GFP vector into mouse primary hippocampal neurons. Three days later, neurons were treated with diosgenin (1 µM) or vehicle solution for 4 days. **H** The expression level of SPARC and **I** pNF-H-positive axonal length in GFP^+^ neurons (green arrowheads) were quantified in each group. Blue rectangles indicate high magnification images of GFP^+^ neurons. *p < 0.05, ***p < 0.001; ^#^*p* < 0.05, ^####^*p* < 0.0001 vs siControl/Veh or siControl/Dios, two-tailed unpaired *t*-test, mean ± standard error. **H** *n* = 30–51 neurons, siControl: *r* = 0.214, 1 − *β* = 0.546, siSPARC: *r* = 0.302, 1 − *β* = 0.681. (**I**) *n* = 18–24 photos, siControl: *r* = 0.565, 1 − *β* = 0.986, siSPARC: *r* = 0.256, 1 − *β* = 0.412.

To investigate the gene expression profile of axon-growing neurons in vivo, laser capture microdissection (LCM) was performed to collect individually naive and axon-growing neurons from the CA1 and CA3 of vehicle- (*n* = 3) or diosgenin-treated (*n* = 3) 5XFAD mice (Fig. 2A). Due to scanty neurons for each mouse, neurons from three mice were combined for DNA microarray. The total number of collected neurons was 660 naive neurons and 720 axon-regenerated neurons from brain sections of three mice.

Genes with stable expression levels (log2 > 8) were analyzed using transcriptome analysis console (Thermo Scientific). Hierarchical clustering and scatter plot analysis revealed a striking difference in the expression patterns (fold change > 5 or < −5) between the two neuron types (Fig. 2B, C). In total, 67 genes in the axon-growing neurons were 5-fold up- or downregulated compared with naive neurons (Supplementary Table 1). The database of all the genes is shown in Supplementary Table 2. We focused on the gene with the highest expression level in axon-growing neurons, secreted protein acidic and rich in cysteine (SPARC). The SPARC protein level was confirmed using HPC neuron lysates. Diosgenin treatment for 4 days significantly enhanced the expression of SPARC in cultured HPC neurons (Fig. 2D, Supplementary Fig. 5). In addition, expression level of SPARC in CA1 neurons was significantly lower in 5XFAD than wild-type mice, whereas it was significantly increased by 14-day-diosgenin administration in 5XFAD mice (Fig. 2E, F).

To investigate whether SPARC upregulation is necessary for diosgenin-induced axonal growth, a SPARC knockdown experiment was performed using siRNA transfection. To distinguish the siRNA-transfected neurons, GFP vectors were mixed together with siRNAs, and the expression of SPARC and axonal length were measured only in GFP^+^ neurons. Transfection of 30 nM siRNA for SPARC (siSPARC) for 3 days significantly reduced SPARC expression without affecting axonal growth in HPC neurons (Supplementary Fig. 6A–C). Three days after siRNA transfection, neurons were treated with vehicle or diosgenin for another 4 days (Fig. 2G). As results, upregulation of SPARC mediated by diosgenin was significantly inhibited in siSPARC-transfected neurons (Fig. 2H). In addition, diosgenin-induced axonal growth was completely diminished by SPARC knockdown (Fig. 2I), indicating that SPARC is an essential molecule for diosgenin-induced axonal growth.

### SPARC overexpression recovers memory deficits and promotes axonal growth in 5XFAD mice

**Fig. 3:**
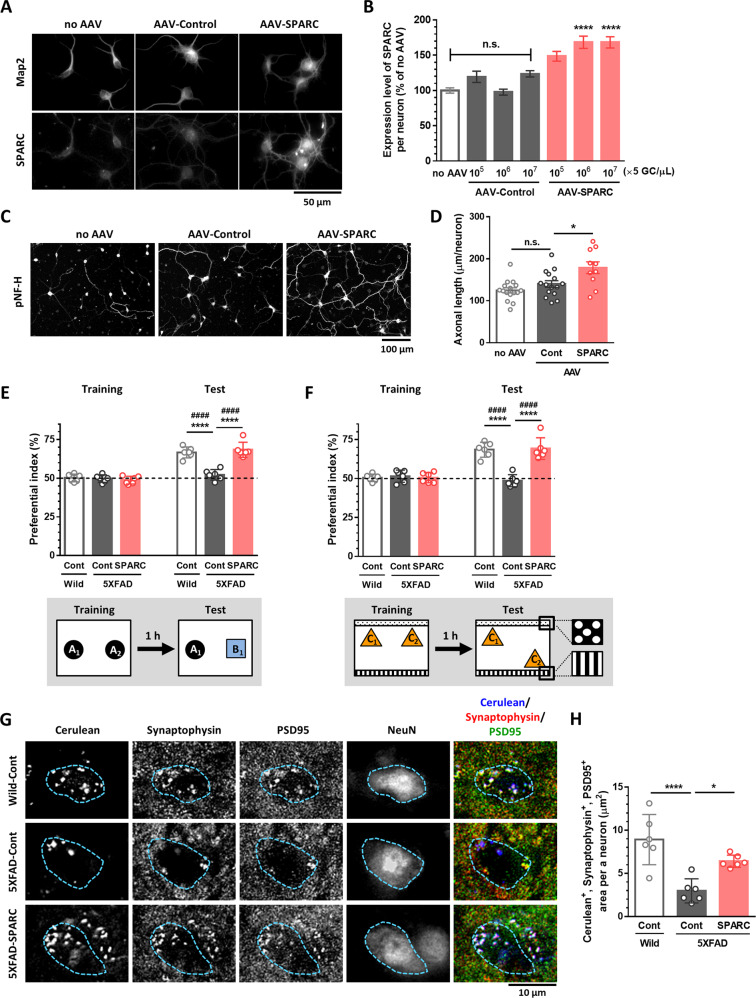

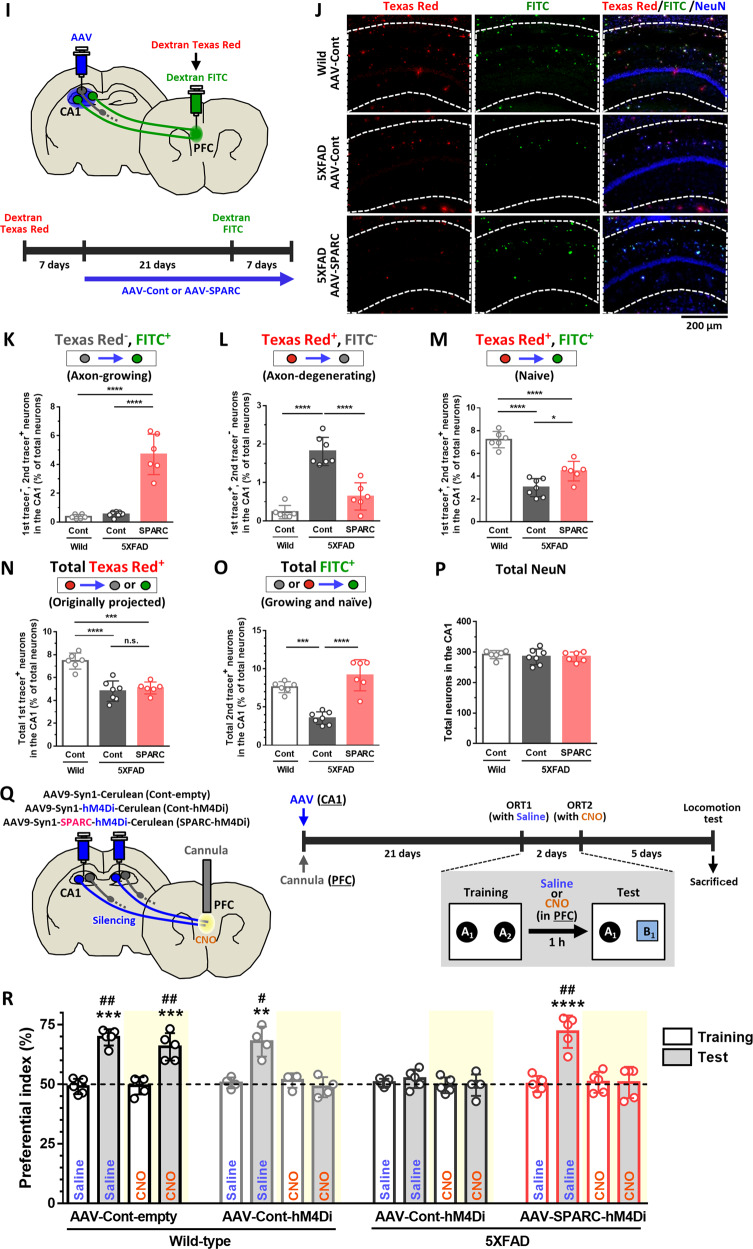
Overexpression of SPARC in the hippocampal neurons recovers memory deficits and promotes axonal growth in 5XFAD mice. Mouse primary hippocampal neurons were treated with 5 × 10^5^, 10^6^, or 10^7^ GC/µl (**A, B**) or 5 × 10^6^ GC/µl (**C, D**) of AAV-Control (AAV9-*Syn1*-Cerulean-WPRE) or AAV-SPARC (AAV9-*Syn1*-mSparc-IRES-Cerulean-WPRE) for 7 days. **A**, **B** SPARC expression levels in MAP2-positive neurons were quantified for each neuron. ****p < 0.0001 vs same concentration of AAV-Control, one-way ANOVA *post-hoc* Bonferroni test, mean ± standard error, *n* = 337–558 neurons. Effect size (*r*) = 0.193, power (1 − *β*) = 1. **C**, **D** pNF-H-positive axon length was quantified for each treatment. *p < 0.05, one-way ANOVA *post-hoc* Bonferroni test, mean ± standard error, *n* = 10–16 photos. *r* = 0.560, 1 − *β* = 0.879. **E**–**H** Wild-type and 5XFAD mice were injected with 10^10^ GC of AAV-Control or AAV-SPARC in the hippocampal CA1 region. Novel object recognition test was performed at 21 days (**E**) and object location test was performed at 23 days (**F**) after AAV injections. The preferential indexes of the training and test sessions are shown. ****p < 0.0001, one-way ANOVA *post-hoc* Bonferroni test. A significant drug × test interaction was found using repeated-measures two-way ANOVA [*F*(2, 15) = 24.30, *p* < 0.0001 (**E**), [*F*(2, 15) = 35.74, *p* < 0.0001 (**F**), ^####^*p* < 0.0001, *post-hoc* Bonferroni test, mean ± standard deviation, *n* = 6 mice/group. **E** *r* = 0.893, 1 − *β* = 0.874, **F** *r* = 0.891, 1 − *β* = 0.871. **G, H** 25 days after AAV injection, colocalization of Cerulean^+^ axons, synaptophysin^+^ pre-synapse, and PSD95^+^ post-synapse on NeuN^+^ neurons (blue dotted line) in the PFC were quantified in each group. *p < 0.05, ****p < 0.0001, one-way ANOVA *post-hoc* Bonferroni test, mean ± standard deviation, *n* = 6 mice/group. **H** *r* = 0.814, 1 - *β* = 0.803. **I**–**P** Seven days after Dextran Texas Red (1st tracing) was injected into the PFC of wild-type and 5XFAD mice, 10^10^ GC of AAV-Control or AAV-SPARC was injected into the hippocampal CA1 region. After 21 days, Dextran FITC (2nd tracing) was further injected into the PFC. Seven days after the 2nd tracer injection, the number of axon-growing (Texas Red^-^, FITC^+^, NeuN^+^) neurons (**K**), axon-degenerating (Texas Red^+^, FITC^−^, NeuN^+^) neurons (**L**), naive (Texas Red^+^, FITC^+^, NeuN^+^) neurons (**M**), originally projected (Texas Red^+^, NeuN^+^) neurons (**N**), axon-growing and naive (FITC^+^, NeuN^+^) neurons (**O**), NeuN^+^ neurons (**P**) in the hippocampal CA1 region were quantified. *p < 0.05, ***p < 0.001, ****p < 0.0001, one-way ANOVA *post-hoc* Bonferroni test, mean ± standard deviation, *n* = 6–7 mice/group. **K** *r* = 0.938, 1 − *β* = 0.921, **L** *r* = 0.921, 1 − *β* = 0.913, **M** *r* = 0.923, 1 − *β* = 0.915, **N** *r* = 0.866, 1 − *β* = 0.876, **O** *r* = 0.895, 1 − *β* = 0.897, **P** *r* = 0.186, 1 − *β* = 0.093. **Q** Wild-type and 5XFAD mice were injected with 10^10^ GC of AAV-Cont-empty, AAV-Cont-hM4Di, or AAV-SPARC-hM4Di in the hippocampal CA1. At the same time, a cannula was infused into the center position covering the right and left PFC. **R** Novel object recognition test was performed at 21 days (microinjected with 0.3 µl saline in the PFC) and 23 days (microinjected with 0.3 µl 1 mM CNO in the PFC) after AAV injections. The preferential indexes of the training and test sessions are shown. **p < 0.01, ***p < 0.001, ****p < 0.0001, one-way ANOVA *post-hoc* Bonferroni test. A significant drug × test interaction was found using repeated-measures two-way ANOVA [*F*(3, 15) = 19.02, *p* < 0.0001 (Saline), [*F*(3, 15) = 10.30, *p* = 0.0006 (CNO). ^#^*p* < 0.05, ^##^*p* < 0.01, *post-hoc* Bonferroni test, mean ± standard deviation, *n* = 4–5 mice/group. Saline: *r* = 0.931, 1 − *β* = 0.980, CNO: *r* = 0.793, 1–*β* = 0.913.

To investigate a direct contribution of SPARC upregulation on axonal growth and memory recovery, the SPARC gene was overexpressed neuron-specifically by adeno-associated virus serotype 9 (AAV9) delivery. AAV9 were engineered to express Cerulean-tagged SPARC, or Cerulean control under a *Syn1* promotor. At first, SPARC overexpression was induced in vitro to test axonal growth in cultured HPC neurons. Seven days after AAV-SPARC treatment, SPARC expression was significantly increased at doses of more than 5 × 10^6^ GC/µl (Fig. 3A, B). At this time, SPARC overexpression (5 × 10^6^ GC/µl AAV-SPARC treatment) significantly increased the axonal length in neurons (Fig. 3C, D).

Next, we investigated whether SPARC overexpression in HPC neurons contributes to memory recovery in 5XFAD mice. At 14, 21, and 28 days post-AAV-SPARC injection to the HPC, SPARC expression was significantly increased in NeuN-positive neurons compared with that in AAV-control-treated mice (Supplementary Fig. 7A–C). Wild-type or 5XFAD mice were injected with AAVs in the CA1 region, and memory tests were performed from 21 days later. In object recognition memory test (Fig. 3E), all mice showed similar exploratory behaviors toward the two identical objects A_1_ and A_2_ during training. In the test session, AAV-SPARC-injected 5XFAD mice showed a significantly higher preferential index to the novel object B_1_ while AAV-control-injected 5XFAD mice did not. To test spatial memory, an object location test was performed 23 days after AAV injection (Fig. 3F). SPARC-overexpressed 5XFAD mice showed a significant improvement in the object location memory compared with AAV-control-injected 5XFAD mice. The total distance traveled (cm), turn angle (degrees), and immobility time (s) were not significantly different between the groups in a locomotion test on day 24 (Supplementary Fig. 8A–C). No significant changes in body weight were observed in any groups (Supplementary Fig. 8D). Taken together, SPARC overexpression in the HPC neurons recovers memory impairment in 5XFAD mice. Twenty-five days after AAV injection, these mice were served to confirm SPARC expression. Similar with Fig. 2E, expression level of SPARC in HPC neurons was significantly lower in 5XFAD than wild-type mice; however, it was significantly increased by AAV-SPARC injection in 5XFAD mice (Supplementary Fig. 8E, F), showing that SPARC overexpression in the HPC was maintained during a series of behavioral tests. Furthermore, synapse formation from the HPC to the PFC were evaluated in these mice (Fig. 3G). Since axons of HPC neurons would be labeled with AAV-derived Cerulean, colocalization of Cerulean, synaptophysin^+^ pre-synapse, and PSD95^+^ post-synapse on NeuN^+^ neurons (blue dotted line) in the PFC were calculated. As a result, synapse formation was significantly decreased between HPC neuron’s pre-synapse and PFC neuron’s post-synapse in 5XFAD compared with wild-type mice; however, SPARC overexpression significantly increased synapse formation in this circuit (Fig. 3H).

Next, we investigated whether overexpression of SPARC in the HPC neurons promotes axonal growth in 5XFAD brains using similar methods as Fig. 1. Dextran Texas Red was injected into the PFC and AAVs were injected into the CA1 7 days later. Twenty-one days after AAV injections, Dextran FITC was injected into the PFC region (Fig. 3I, J). Axonal projection from the CA1 to the PFC was significantly increased in AAV-SPARC-injected 5XFAD mouse brains (Fig. 3K). The number of axon-degenerating neurons (Texas Red^+^, FITC^-^) was significantly rescued by SPARC overexpression (Fig. 3L). The number of naive neurons (Texas Red^+^, FITC^+^) was also increased by SPARC overexpression in 5XFAD mice (Fig. 3M). The originally projected axons (total Texas Red^+^) in AAV-control and AAV-SPARC-injected 5XFAD mice were not different, showing that the same degree of axonal degeneration had occurred in these 5XFAD mice before AAV injections (Fig. 3N). The number of total FITC^+^ neurons was significantly increased by SPARC overexpression with equal level as wild-type mice (Fig. 3O). The number of total NeuN^+^ neurons did not differ among the groups (Fig. 3P). A significant increase in axonal growth and decrease in axonal degeneration from the CA3 to the PFC were observed by SPARC overexpression in 5XFAD mice compared with AAV-control-injected mice (Supplementary Fig. 9). These data suggested that SPARC overexpression in the HPC neurons enhanced long-distance axonal projection in 5XFAD mouse.

### SPARC overexpression-induced axonal growth contributes to memory recovery in 5XFAD mice

To confirm direct contributions of SPARC-driven axonal growth on memory recovery, DREADDs [20] was used to silence neural activity of SPARC-overexpressed axons projected from the HPC to the PFC. AAV9 was engineered to express Cerulean-tagged SPARC with inhibitory muscarinic receptor variant hM4Di (AAV-SPARC-hM4Di) under a *Syn1* promotor to overexpress both SPARC and hM4Di in neurons. hM4Di-expressing neurons respond to clozapine-N-oxide (CNO) to activate Gi signaling. Therefore, AAVs were injected into the HPC, and CNO was microinjected through a cannula inserted in the PFC.

At 21 and 28 days post-AAV-SPARC-hM4Di injection to the CA1, SPARC expression was significantly increased in NeuN-positive neurons (Supplementary Fig. 10). Additionally, AAV-derived hM4Di (Cerulean) was observed in the PFC at the location where the axons of HPC neurons terminate (Supplementary Fig. 10). A cannula for the CNO infusion was inserted in the PFC (Fig. 3Q). Object recognition memory tests were performed 21 and 23 days after AAV injection immediately after microinjection of saline or 1 mM CNO, which is the reported dose with no abnormal behaviors by itself [21]. Microinjection of saline in the PFC did not interfere with memory retention in wild-type mice and AAV-SPARC-hM4Di-injected 5XFAD mice (Fig. 3R). Microinjection of CNO had no influence on the memory function of hM4Di-absent AAV-Cont-empty-injected wild-type mice. However, CNO injection impaired object recognition memory in AAV-Cont-hM4Di-injected wild-type mice, showing that the HPC-PFC circuit is indispensable to memory formation. Importantly, when CNO was microinjected into the PFC of AAV-SPARC-hM4Di-injected 5XFAD mice, SPARC overexpression-induced memory recovery was completely diminished. These results indicate that SPARC overexpression-induced axonal growth directly contributes to memory recovery in 5XFAD mice.

The results of the locomotion test on day 28 showed that total distance traveled (cm), turn angle (degrees), and immobility time (s) were not significantly different between the groups (Supplementary Fig. 11A–C). The cannula was located in identical positions within the PFC (Supplementary Fig. 11D). SPARC overexpression was maintained in the CA1 neurons of AAV-SPARC-hM4Di-injected 5XFAD mice, and hM4Di was observed in the axons of HPC neurons terminating to the PFC in all groups (Supplementary Fig. 11E, F).

### SPARC expression is increased on the axonal area

**Fig. 4:**
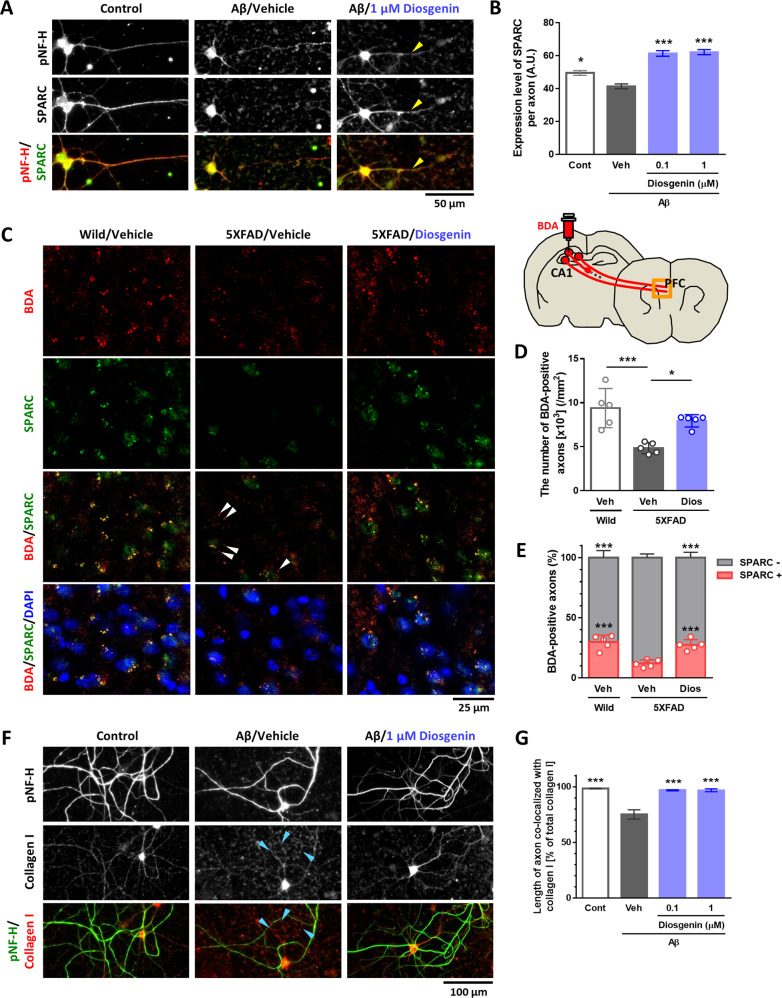

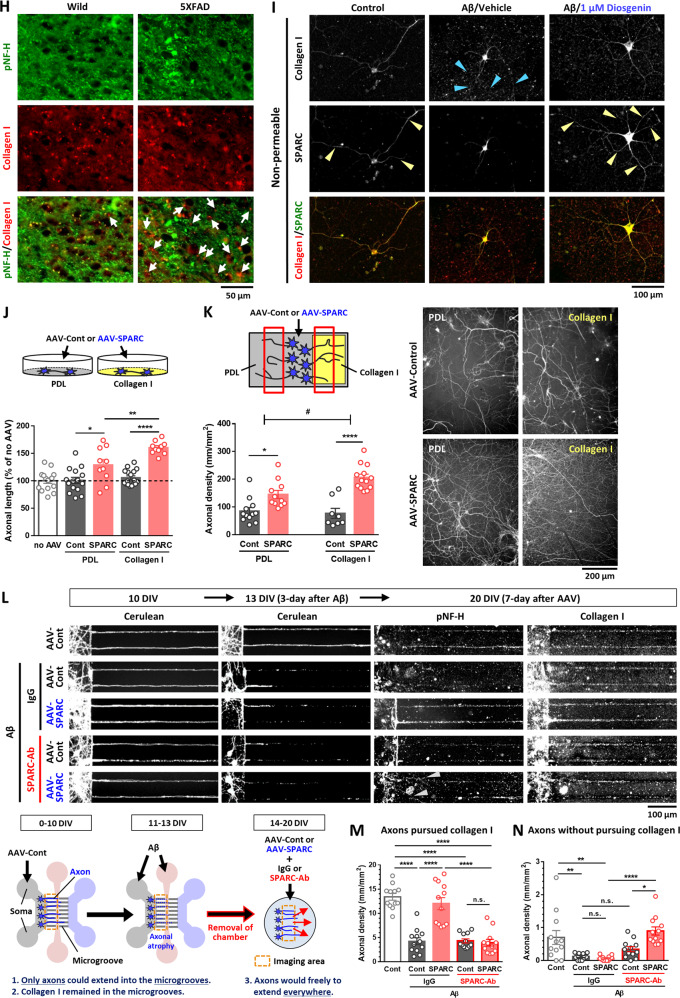
Interaction of SPARC on axonal membranes with extracellular collagen I is required for axonal remodeling. **A**, **B** Mouse primary hippocampal neurons were cultured for 3 days and then treated with or without Aβ_25-35_ (2.5 µM) for 3 days. Next, neurons were treated with diosgenin (0.1 or 1 µM) or vehicle solution for 4 days. The SPARC level was increased particularly on axonal shafts (yellow arrowheads) in diosgenin-treated neurons (**A**). SPARC expression level on axons was measured for each treatment (**B**). *p < 0.05, ***p < 0.001 vs Aβ_25-35_ (Aβ)/Vehicle (Veh), one-way ANOVA *post-hoc* Bonferroni test, mean ± standard error, *n* = 92–427 axons. Effect size (*r*) = 0.248, power (1 − *β*) = 1. **C**–**E** Wild-type and 5XFAD mice were administered diosgenin (Dios; 0.1 µmol/kg/day) or vehicle solution (Veh) for a total of 21 days. On administration day 14, anterograde tracer BDA was injected into the hippocampal CA1 region. After 7 days, BDA-positive axons (red), SPARC expression (green), and DAPI (blue) were detected in the PFC (**C**). The number of BDA-positive axons (**D**) and percentage of SPARC-positive and BDA-positive or SPARC-negative and BDA-positive axons (**E**) was measured for each mouse. *p < 0.05, ***p < 0.001 vs 5XFAD/Veh, one-way ANOVA *post-hoc* Bonferroni test, mean ± standard deviation, n = 5 mice/group. **D** *r* = 0.835, 1 − *β* = 0.722, **E** *r* = 0.887, 1 − *β* = 0.776. **F**, **G** Mouse primary hippocampal neurons were cultured for 14 days and treated with Aβ_25-35_ (2.5 µM) for 3 days, then with diosgenin (0.1 or 1 µM) or vehicle solution for 4 days. **F** Dot-like traces of collagen I were observed along degenerated axons in Aβ-treated neurons (blue arrowheads). **G** Length of axons colocalized with collagen I (pNF-H^+^, collagen I^+^) was measured in each group. ***p < 0.001 vs Aβ/Veh, one-way ANOVA *post-hoc* Bonferroni test, mean ± standard error, *n* = 12 images/group. *r* = 0.951, 1 − *β* = 0.999. **H** Immunohistochemistry detected pNF-H-positive axons (green) and collagen I (red) in the PFC of wild-type and 5XFAD mice. Collagen I-positive but pNF-H-negative axons was often observed in 5XFAD mice (white arrows). **I** Mouse primary hippocampal neurons were cultured for 14 days and treated with Aβ_25–__35_ (2.5 µM) for 3 days, then with diosgenin (0.1 or 1 µM) or vehicle solution for 4 days. SPARC on plasma membranes and extracellular collagen I were detected by antibodies in non-permeable immunocytochemistry. SPARC on axonal membranes and its colocalization with collagen I were observed in control and Aβ/Diosgenin groups (yellow arrowheads). Collagen I was observed along degenerated-axons in Aβ-treated neurons (blue arrowheads). **J** Mouse primary hippocampal neurons were cultured on PDL- or collagen I-coated dishes. AAV-Control or AAV-SPARC (5 × 10^6^ GC/µl) was treated for 7 days, and pNF-H-positive axonal lengths were quantified. *p < 0.05, **p < 0.01, ****p < 0.0001, one-way ANOVA *post-hoc* Bonferroni test, mean ± standard error, *n* = 10–17 images/group, *r* = 0.740, 1 − *β* = 0.999. **K** PDL and collagen I were coated on the left and right side, respectively. AAV-Control- or AAV-SPARC (5 × 10^6^ GC/µl)-treated mouse primary hippocampal neurons were seeded on PDL-coated center part. pNF-H-positive axonal lengths were quantified after 14 days. *p < 0.05, ****p < 0.0001, one-way ANOVA; ^#^*p* < 0.05, two-way ANOVA, *post-hoc* Bonferroni test, mean ± standard error, 8–13 images/group, *r* = 0.765, 1 − *β* = 0.989. A significant SPARC × collagen I interaction was found using repeated-measures two-way ANOVA [F(1, 40) = 5.81, *p* = 0.0206]. **L**–**N** Mouse primary hippocampal neurons were seeded on the soma space (gray) of a triple chamber neuron device and treated with 5 × 10^7^ GC/µl AAV-control for 10 days. Cerulean-labeled axons in the microgrooves were observed using live cell imaging on 10 DIV (days in vitro). After that, Aβ_25–__35_ (2.5 µM) was treated to soma (gray) and axonal space (pink) for 3 days. Live cell imaging in 13 DIV confirmed Cerulean-labeled axons that originally extended into the microgrooves were atrophied by Aβ_25–__35_. Then, triple chamber neuron devices were removed, and 5 × 10^6^ GC/µl AAV-control or AAV-SPARC were treated together with 2 µg/ml SPARC neutralizing antibody (SPARC-Ab) or control IgG (IgG). Densities of **M** axons pursued extracellular collagen I and **N** axons without pursuing extracellular collagen I were quantified at 20 DIV in each group. *p < 0.05, **p < 0.01, ****p < 0.0001, one-way ANOVA, *post-hoc* Bonferroni test, mean ± standard error, 12 images/group, **M** *r* = 0.847, 1–*β* = 0.999, **N** *r* = 0.644, 1 − *β* = 0.982.

We investigated the intracellular localizations of SPARC in diosgenin-treated neurons compared with normal control and Aβ_25–__35_-treated neurons. HPC neurons were treated with Aβ_25–__35_ for 3 days, and diosgenin or vehicle solution was applied for 4 days (Fig. 4A). Immunostaining detected intrinsic expression of SPARC in neuronal bodies and axonal shafts in the control neurons, whereas Aβ treatment significantly decreased the SPARC expression, particularly in axons (Fig. 4B). However, diosgenin treatment significantly increased the expression of SPARC on axons compared with Aβ_25–__35_/vehicle groups. Since we have previously identified 1,25D_3_-MARRS as a direct binding receptor of diosgenin to induce axonal regrowth and memory recovery [9, 10, 14], we investigated whether SPARC upregulation on axons was mediated by 1,25D_3_-MARRS signaling. Three days after culturing HPC neurons, Aβ_25–__35_ was treated for 3 days followed by 1,25D_3_-MARRS neutralizing antibody or control IgG and vehicle or diosgenin treatments for 4 days. As results, diosgenin-induced axonal regrowth and SPARC upregulation were diminished by 1,25D_3_-MARRS neutralizing antibody treatment (Supplementary Fig. 12A, B), suggesting that that diosgenin-induced upregulation of SPARC on axons was mediated by 1,25D_3_-MARRS signaling.

The expression of SPARC on axons from the HPC to the PFC was investigated by anterograde tracing of biotinylated dextran amines (BDA). Vehicle solution or diosgenin was administered daily for 21 days, and on administration day 14, BDA was injected in the CA1 (Fig. 4C). Injected BDA colocalized with hypophosphorylated NF-H (pNF-H)-positive axons in the PFC (Supplementary Fig. 13). The quantitative values of BDA-positive axons in the PFC were significantly reduced in vehicle-treated 5XFAD mice compared with wild-type mice (Fig. 4D). However, diosgenin administration significantly increased axonal projection to the PFC. In the PFC of wild-type mice, high expression of SPARC on BDA-positive axons was observed (Fig. 4C). However, the number of SPARC-expressing axons in the 5XFAD PFC was significantly decreased (Fig. 4E), and a large part of BDA-positive axons did not express SPARC (Fig. 4C, white arrowheads). When 5XFAD mice were treated with diosgenin, SPARC-expressing BDA-positive axons significantly increased to the equal level as that in wild-type mice. These data indicated that upregulation of SPARC was significant in axonal terminal area by diosgenin treatment.

### Elevated axonal SPARC levels induce accurate pathfinding of injured axons

Based on evidence showing high expression of SPARC in regrowing axons, we speculated that accurate pathfinding of injured axons brought by SPARC elevation may require an interacting extracellular counterpart molecule for arrival at the terminating site. SPARC consists of three domains, namely the acidic, follistatin-like, and extracellular domains [22], and is expressed in the cytoplasm [23], extracellular matrix [24, 25], and plasma membrane [26]. Regarding SPARC located on axonal membranes, SPARC possibly interact with extracellular molecules via its extracellular domain. One of the main ligands of the extracellular domain of SPARC is the collagens; [27] collagen types I, III, and IV were reported. The collagen family promotes axonal growth, axonal guidance, and synapse formation [28]. However, the effect of the interaction of collagen with SPARC on axonal formation has never been studied. Since the expression of collagen type I (collagen I) is high in the brain, SPARC-collagen I interaction on axonal regeneration was investigated.

Short-term culture of HPC neurons could rarely induce the expression of collagen I, whereas its expression was increased in long-term-cultured neurons (Supplementary Fig. 14A, B). Therefore, HPC neurons were cultured for 14 days and treated with Aβ_25–__35_ for 3 days followed by 4-day treatment with diosgenin. In control neurons, collagen I was distributed in a fiber-like shape and colocalized with axons (Fig. 4F). However, some collagen I existed like axonal wreckages and did not colocalize with axons in Aβ_25–__35_-treated neurons (blue arrowheads). Since collagen I is produced and secreted from neurons [29, 30], we speculated that this neuron-derived collagen I remained in the extracellular space even after axonal disruption. Quantitative graphs showed that length of pNF-H^+^ axons that colocalize with collagen I-positive fibers was significantly decreased by Aβ_25–__35_ treatment compared with control neurons; however, colocalization of axons with collagen I-positive fiber significantly increased by diosgenin (Fig. 4G). SPARC signal was not detected when only secondary antibody for SPARC were used for immunostaining (Supplementary Fig. 14C). In addition, a negative control peptide for Aβ_25-35_, Aβ_35-25_, did not influence on levels of colocalization of axons and collagen I (Supplementary Fig. 14D) and expression of SPARC on axons (Supplementary Fig. 14E). A significant colocalization of axons with collagen I was also detected in the PFC of wild-type mice (Fig. 4H); however, collagen I-positive but pNF-H-negative dots were increased in 5XFAD mice (white arrows). Furthermore, SPARC on axonal membranes and its colocalization with extracellular collagen I were confirmed by non-permeable immunocytochemistry (Fig. 4I). SPARC signals were detected on axonal membranes (yellow arrowheads) and colocalized with extracellular collagen I in control neurons. In Aβ_25-35_-treated neurons, the expression of SPARC on axonal membranes was drastically decreased, and extracellular collagen I alone remained (blue arrowheads). However, diosgenin treatment increased the expression of SPARC on axonal membranes, colocalizing with collagen I (yellow arrowheads). These results led us to hypothesize that even if axons were degenerated by Aβ, extracellular collagen I still be located on their original tracks; therefore, direction-specific axonal regeneration may occur when elevated levels of SPARC on axonal membranes interact with extracellular collagen I.

To test this hypothesis, the effect of SPARC-collagen I interaction on axonal growth was investigated. Prior to culturing the neurons, collagen I was overlaid on poly-D-lysine (PDL)-coated dishes, and HPC neurons were then treated with AAV-control or AAV-SPARC 4 h after culturing (Fig. 4J). Axonal length was significantly increased by 7-day-SPARC overexpression in the PDL-coated dish. Interestingly, the SPARC-driven axonal growth was significantly increased further by collagen I coating. These data indicated that the SPARC-collagen I interaction promotes axonal growth.

To investigate whether extracellular collagen I relates to accurate pathfinding of axons in SPARC-overexpressed neurons, collagen I was coated in direction-limited (Fig. 4K). Collagen I was overlaid on the right side of the dish, and HPC neurons were seeded on a collagen I-free space using device chambers. After removal of device chambers, AAVs were treated for 14 days. Axons growing onto the PDL- and collagen I-coated regions were quantified. AAV-control-treated neurons showed similar axonal growth preference to the PDL and collagen I. Meanwhile, axonal growth of SPARC-overexpressed neurons showed a directivity toward collagen I.

Furthermore, we tested whether interaction of SPARC on axonal membranes and extracellular collagen I actually promoted directional regrowth of axons (Fig. 4L). HPC neurons were seeded on the soma space (gray) of a triple chamber neuron device and treated with 5 × 10^7^ GC/µl AAV-control to visualize axons by Cerulean fluorescence in live imaging. Since only axons could extend into microgrooves of the device chambers, neuron-derived extracellular collagen I would remain in the microgrooves where axons originally elongated. Ten days after neuron culture, Cerulean^+^ axons were observed using a fluorescence microscope to confirm axonal extension into the microgrooves. Then, Aβ_25–__35_ was treated to soma (gray) and axonal space (pink) for 3 days (11–13 DIV [days in vitro]). Live imaging in 13 DIV revealed that exactly same Cerulean^+^ axons that originally extended into the microgrooves were atrophied by Aβ_25–__35_ treatment. After that, triple chamber neuron devices were removed from the bottom of the dishes, and 5 × 10^6^ GC/µl AAV-control or AAV-SPARC were treated together with SPARC neutralizing antibody (SPARC-Ab) or control IgG (IgG). Since device chambers were removed, axons would freely to regrow everywhere. Axonal densities in the original microgroove space (axons pursued extracellular collagen I) or out of microgroove space (axons did not pursue extracellular collagen I) were quantified in 20 DIV. As results, SPARC overexpression significantly regrew axons to pursue collagen I in control IgG-treated group (Fig. 4M). On the other hand, axonal regrowth into collagen I direction was completely inhibited by SPARC-Ab treatment, suggesting that membranal SPARC was required for axons to regrow into their original tracks. Interestingly, as we expected, density of axons without pursuing collagen I (gray arrowheads) was significantly increased by SPARC-Ab treatment in SPARC-overexpressed neurons (Fig. 4N), which failed to induce direction-specific axonal regrowth. Extracellular collagen I was existed exactly along the axons ran in the microgroove area in all groups even after axonal atrophy (Fig. 4L). All images were captured in exact same region throughout all time points in each group. These data together demonstrated that elevated levels of SPARC on axonal membranes interact with the guidepost molecule, extracellular collagen I, resulting in accurate axonal regrowth.

## Discussion

**Fig. 5:**
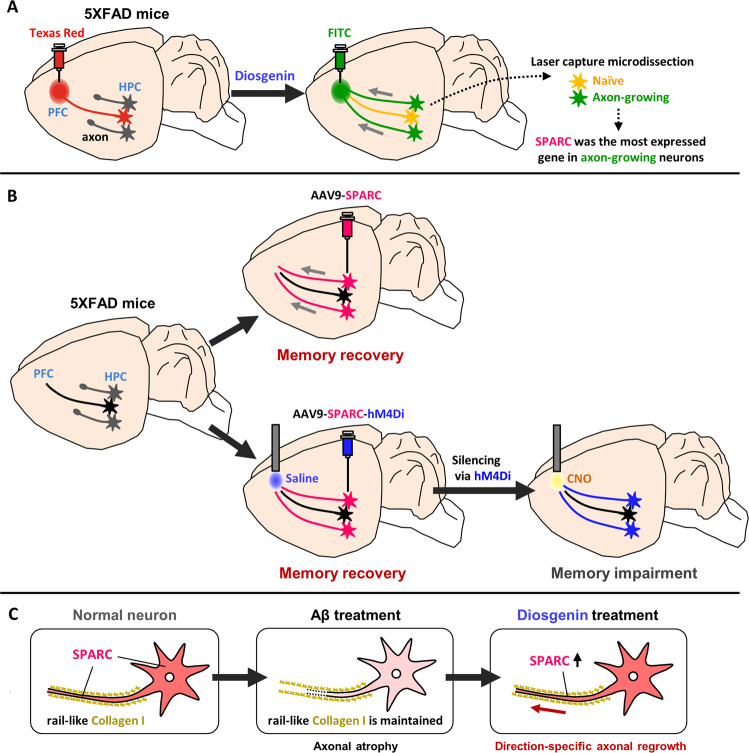
Study summary: Diosgenin-induced axonal growth and corresponding molecular mechanisms in 5XFAD mice brain. **A** Diosgenin administration promoted axonal growth from the hippocampus (HPC) to the prefrontal cortex (PFC) in 5XFAD mice. Secreted protein acidic and rich in cysteine (SPARC) was the most expressed molecule in the axon-growing neurons. **B** Overexpression of SPARC (by AAV9 injection) in 5XFAD HPC (without diosgenin administration) recovered memory function and axonal regeneration in this neural circuit. When SPARC overexpression-driven axonal growth from the HPC to the PFC was silenced by DREADDs, memory recovery of 5XFAD mice was diminished. **C** SPARC interacted with extracellular rail-like collagen I which remained in the place where axons were originally located. Elevated SPARC, especially on axonal membranes, interacted with the guidepost collagen I, resulting in accurate axonal regeneration in primary cultured HPC neurons.

We demonstrated that degenerated axons re-innervated in a long-distance to their target regions in AD model mouse brains. SPARC was identified as a critical molecule in neurons in regulating axonal regrowth. The interaction of SPARC on axons with extracellular collagen I is a novel molecular mechanism for controlling the accurate pathfinding of injured axons (Fig. 5). These findings indicate a promising therapeutic strategy to restore axons for AD treatment.

Although transected axons of motor neurons regrow toward their original termination sites via *Ih3* glycosyltransferase in the peripheral nervous system [31], it remains undetermined as to how atrophied axons in the CNS reach their native projecting area. To investigate molecular mechanisms underlying axonal regrowth in the 5XFAD mouse brain, we individually isolated axon-growing neurons from diosgenin-treated 5XFAD mouse brains using LCM to compare their gene expression with that in naive neurons (Fig. 2A). LCM is a technique capable of isolating different phenotypes of cells even from the same confined tissue. Although neurons from three mice were combined for DNA microarray due to scanty neurons in each mouse, the expression profiles of axon-regenerated and naive neurons were comparatively different. SPARC showed 15.95 times higher expression of mRNA than that in naive neurons, and its protein level was also significantly increased by diosgenin treatment in vitro (Fig. 2D) and in vivo (Fig. 2E, F), suggesting the high accuracy of LCM and gene profiling for identifying differently expressed molecules based on single-cell phenotype. In addition, importantly, we confirmed that SPARC upregulation was actually involved in diosgenin-induced axonal growth using SPARC knockdown experiment (Fig. 2G–I).

SPARC is a glycoprotein that promotes cell adhesion [32, 33], proliferation [34], migration [35], tissue renewal and repair [36], and development [37]. In the development of the CNS, the expression of SPARC peaks during neuronal circuit formation [38], and SPARC participates in synapse formation [39] and removal [40]. Interestingly, SPARC is increased in the HPC after transection of the entorhinal cortex, one of the axonal terminal sites of HPC neurons [41], showing the possibility that SPARC contributes to neural repair. However, it remains unclear whether SPARC upregulation promotes axonal growth. We showed that overexpression of SPARC in the HPC neurons repaired axons toward PFC and recovered memory deficits in 5XFAD mice.

SPARC may be a key modulator for controlling axonal pathfinding by interacting with extracellular collagen I that localizes like guidepost molecules on the original axon tracings. Since collagen I is produced in cultured neurons [29, 30], the existence of collagen I in the neuronal cultures was unsurprising (Fig. 4F). However, collagen I remained on axonal tracking even after axonal shafts were disrupted by Aβ. SPARC was expressed on axonal membranes, and SPARC-overexpressed neurons enhanced directional axonal growth in the presence of collagen I. These results were consistent with a previous study showing that neurite growth of dorsal root ganglion neurons was enhanced in the presence of extracellular collagen I [42].

We clarified that diosgenin treatment extended axons from the HPC to the PFC in 5XFAD mice. The diosgenin-driven accurate pathfinding was explained by SPARC upregulation. This illustrates the effectiveness of diosgenin as an activator of re-innervation of axons and attractive candidate of AD drug. In the present study, we showed that diosgenin-induced SPARC upregulation was mediated by 1,25D_3_-MARRS, a direct binding protein for diosgenin in neurons (Supplementary Fig. 12). It is known that one of the transcriptional factors for SPARC is c-Jun [43]. We have previously clarified that diosgenin at least activates phosphoinositide 3-kinase, extracellular signal-regulated kinase, protein kinase A, and protein kinase C to induce axonal growth [10]. Since the transcriptional activity of c-Jun is enhanced by activation of these four protein kinases [44], we speculated that diosgenin may upregulate the expression of SPARC via transcriptional activity of c-Jun.

Diosgenin-induced upregulation of SPARC was about 1.5-fold compared with vehicle in vitro (Figs. 2D) and 1.6–1.7-fold compared with vehicle-treated 5XFAD mice in vivo (Fig. 2F). On the other hand, 5 × 10^6^ GC/µl AAV-SPARC, the dose which significantly induced axonal growth (Fig. 3C, D), treatment increased SPARC level in 1.6–1.7-fold compared with AAV-Cont in vitro (Fig. 3B), and AAV-SPARC injection increased SPARC level in 1.6–1.7-fold compared with AAV-Cont-injected 5XFAD mice in vivo (Supplementary Fig. 8F). Therefore, AAV-SPARC could almost mimic diosgenin-induced SPARC upregulation in this study. However, the level of axonal growth from the HPC to the PFC in 5XFAD mice was slightly higher in SPARC overexpression (Fig. 3K) compared with diosgenin administration (Fig. 1D), suggesting that effects of diosgenin and SPARC overexpression were not completely equal. Since in vivo transcriptome analysis revealed that there were many changed genes other than SPARC in axon-growing neurons (Fig. 2C), future studies will focus on these molecules to investigate synergistic effects and counteracting effects on axonal growth.

Although we have not identified the concrete nature and characteristics of the HPC neurons that projected axons toward the PFC by diosgenin or SPARC overexpression in the present study, axon-growing neurons expressed 5.5-fold higher Calretinin (Calbindin 2) compared with the naive neurons according the result of microarray (Supplementary Table 2). Calretinin is a calcium-binding protein that regulates calcium homeostasis [45], and calcium signaling plays a role in axonal growth and guidance [46]. Importantly, it has been reported that more than half of HPC neurons that project axons to PFC neurons were Calretinin-positive [47], suggesting that Calretinin-positive neurons may be involved in long-distance axonal projection from the HPC to the PFC. Furthermore, the localization pattern of axon-growing neurons (Dextran Texas Red^-^ and FITC^+^) in our present study was very similar to the location of Calretinin-positive neurons in the HPC including the *Stratum oriens*, *S. pyramidale*, *S. radiatum*, and *S. lacunosum moleculare* [48, 49]. Therefore, we speculate that some population of the Calretinin-positive neurons may have ability to extend axons at long distance even after axonal atrophy. Future studies should evaluate the function and characteristics of these axon-growing neurons by electrophysiologic and single-cell analyses.

Memory deficits in 5XFAD mice are primarily caused by neurite degeneration and synaptic dysfunction, but not neuronal death in the brain. Although memory deficits begin at 4–5 months of age in 5XFAD mice, neuronal loss was not observed in this age [8, 50]. Therefore, long-distance axonal growth should be a direct cause for memory recovery in 5XFAD mice. This is the first study to show that axons in AD model mice extend toward a distant target region, in the accurate projecting area, which contributes to memory recovery. Diosgenin is a potential treatment agent to stimulate axonal growth via these mechanisms. Our findings suggest that axons in the brain have the capacity to growth, and that promoting axonal growth potentially comprises a promising therapeutic strategy for AD.

### Supplementary information

Supplementary information

Supplementary Table 1

Supplementary Table 2
