# Supplementary material for: Diosgenin restores memory function via SPARC-driven axonal growth from the hippocampus to the PFC in Alzheimer’s disease model mice
Source: Mol Psychiatry. 2023 Apr 22;28(6):2398–411. doi: 10.1038/s41380-023-02052-9 (PMC10611574; doi:10.1038/s41380-023-02052-9)
Supplement: Supplementary file 1 — Supplementary information [file 41380_2023_2052_MOESM1_ESM.docx]

**SUPPLEMENTARY INFORMATION**

**Diosgenin restores memory function via SPARC-driven axonal growth from the hippocampus to the PFC in Alzheimer’s disease model mice**

Ximeng Yang^1^ and Chihiro Tohda*^1^

^1^Section of Neuromedical Science, Institute of Natural Medicine, University of Toyama, 2630 Sugitani, Toyama 930-0194, Japan

*Correspondence:

Chihiro Tohda, Ph.D.

Section of Neuromedical Science, Institute of Natural Medicine,

University of Toyama, 2630 Sugitani, Toyama 930-0194, Japan

Phone and Fax: +81-76-434-7646

E-mail: [chihiro@inm.u-toyama.ac.jp](mailto:chihiro@inm.u-toyama.ac.jp)

**Materials and methods**

**Mice**

All experiments were performed in accordance with the Guidelines for the Care and Use of Laboratory Animals of the University of Toyama. The Committee for Animal Care and Use in the University of Toyama approved the study protocol (approval number A2020INM-1). All efforts were made to minimize the number of animals used. Since the strain, sex, and age of mice were same in each experiment, the mice were randomly to be allocated to experimental groups. The animal experiments were unblinded to be performed.

Transgenic mice (5XFAD; +/-) and the corresponding wild-type mice (-/-) were obtained from the Jackson Laboratory (Bar Harbor, ME, USA) and maintained by crossing hemizygous animals with B6/SJL F1 breeders. 7–9-month-old female 5XFAD and female wild-type mice were used for experiments. All mice were housed in a controlled environment (25 ± 2 °C, 12-h light/dark cycle starting at 7:00 am) with free access to food and water.

**Axotomy**

ddY mice were purchased from Japan SLC (Shizuoka, Japan). Craniotomy was performed overlying the motor cortex in adult ddY mice (female, 8 weeks old) placed on a stereotaxic device (Narishige, Tokyo, Japan). A miniature blade was slowly advanced into the brain (+0.0 mm anterior-posterior; A-P, +1.5 mm medial-lateral; M-L, -1.6 mm dorsal-ventral; D-V relative to the bregma), and moved parallel to +1.5 mm A-P. During and following surgery, the mice were kept on a hotplate to maintain body temperature and were anesthetized 1 h and 7 days after axotomy or were administered drugs from 7 days after axotomy.

**Diosgenin administration**

Diosgenin compound was purchased from Tokyo Chemical Industry (Tokyo, Japan). Diosgenin (0.1 µmol/kg/day) was dissolved in Japanese Pharmacopoeia quality standard olive oil (Maruishi Pharmaceutical Co., Ltd, Osaka, Japan) or vehicle solution (olive oil), as reported previously [11], and orally administered once a day to axotomized ddY mice and wild-type or 5XFAD mice.

**Retrograde labeling using Dextran 3000 MW**

Craniotomy was unilaterally performed on a stereotaxic device to expose a small section of the PFC in each mouse. 0.5 µL of Dextran 3000 MW Texas Red (50 mg/mL in artificial cerebrospinal fluid [aCSF]; catalog no. D3328, Thermo Fisher Scientific, Waltham, MA, USA) and 0.5 µL of Dextran 3000 MW FITC (50 mg/mL in aCSF; catalog no. D7156, Thermo Fisher Scientific, Waltham, MA, USA) were sequentially injected into the same region of the PFC (+1.9 mm A-P, +0.3 mm M-L, -2.6 mm D-V) at a rate of 0.5 µL/min in 5XFAD and wild-type mice. Seven days after the Dextran 3000 MW Texas Red injection, drug administration or AAV9 injection was performed as described above and below. Subsequently, Dextran 3000 MW FITC was injected into the PFC for 7 days. When the Dextran 3000 MW FITC was injected, a hole in the skull derived from the craniotomy for the Dextran 3000 MW Texas Red injection could be observed; therefore, a careful examination through both the stereotaxic device and eyesight was conducted so that the injection of the second tracer would be performed at the exact same position as that for the first tracer. Only those mice that had a complete match between the positions of injection for the two tracers were included in the final analyses.

**LCM and DNA microarray**

Vehicle- or diosgenin-treated 5XFAD mice were deeply anesthetized with isoflurane and perfused with 20 ml of ice-cold saline. The brains were carefully removed from the skull, quick-frozen using dry ice, and stored at -30 °C. The brains were cut into 16-µm coronal slices using a cryostat (Leica, Heidelberg, Germany), attached on RNase-free glass slides, and stored at -80 °C until LCM. LCM was performed using PALM MicroBeam (Carl Zeiss, Oberkochen, Germany). Neurons from at least one slide were captured within 1 h at room temperature. A total of 660 naïve neurons from three vehicle-treated 5XFAD mice and 720 axon-growing neurons from three diosgenin-treated 5XFAD mice were captured from the brain slices.

Total RNA was isolated from captured cells (NucleoSpin RNA, MACHEREY- NAGEL GmbH & Co. KG, Duren, Germany) and subjected to T7 RNA polymerase amplification (GeneChip™ 3’ IVT Pico Kit, Thermo Scientific). The amplified RNA was hybridized (GeneChip™ Hybridization, Wash and Stain Kit, Thermo Scientific) to the Mouse Clariom S Array. The data were analyzed using the transcriptome analysis console (Thermo Scientific).

**Primary neuron culture and immunocytochemistry**

Embryos were removed from a pregnant ddY mouse (Japan SLC, Shizuoka, Japan) at 14 days of gestation and the hippocampus was isolated, as described previously [10]. Neurons were treated with 5×10^5^, 10^6^, or 10^7^ GC/µl (Fig. 3A, B) or 5×10^6^ GC/µl (Fig. 3C, D and Fig. 4J, K) of AAV-Control or AAV-SPARC for 7 days. For Aβ treatment, Aβ_25-35_ or its negative control peptide Aβ_35-25_ (Sigma-Aldrich, St. Louis, MO, USA) was incubated at 37 °C for 4 days prior to treatment to facilitate aggregation. Neurons were cultured for 3 days and treated with 2.5 μM Aβ_25-35_ for 3 days and then with 0.1 or 1 µM diosgenin or vehicle solution (ethanol) for 4 days (Fig. 4A, B). For long-term cultures, neurons were cultured for 14 days, and treated with 2.5 μM Aβ_25-35_ or Aβ_35-25_ for 3 days and then with 0.1 or 1 µM diosgenin or vehicle solution (ethanol) for 4 days (Fig. 4F, G, I; Supplementary Fig. 14C–E). For 1,25D_3_-MARRS neutralizing antibody treatment in Supplementary Fig. 12, Aβ_25-35_ was treated for 3 days followed by 70 µg/mL anti-rabbit 1,25D_3_-MARRS neutralizing antibody (Ab099 clone, gifted by Dr. Ilka Nemere) or normal rabbit IgG (control IgG) together with 1 µM diosgenin or vehicle solution (ethanol) for 4 days.

Other than Fig. 4I, the neurons were fixed with 4% paraformaldehyde for 90 min and immunostained at 4 °C for 24 h in 0.3% Triton X-PBS with primary antibodies. For antibodies non-permeable experiments in Fig. 4I, the neurons were fixed with 4% paraformaldehyde for 15 min and immunostained at 4 °C for 24 h in Triton X-free PBS with the following primary antibodies: Mouse anti-pNF-H (1:250, Covance, catalog no. SMI 35), rabbit anti-microtubule-associated protein 2 (MAP2; 1:2,000, Abcam, catalog no. ab32454), goat anti-mouse SPARC (1:100, R&D systems, catalog no. AF942), and rabbit anti-collagen type I (1:500, Abcam, catalog no. ab34710) antibody. Secondary antibodies—Alexa 488-, 568-, 594-, or 647-conjugated affinity-purified secondary anti-mouse IgG, anti-rabbit IgG, and anti-goat IgG—were reacted for 2 h at room temperature in 0.3% Triton X-PBS or PBS (Fig. 4J). Staining with 1 µg/ml DAPI (in PBS) was performed at room temperature for 10 min. Fluorescence images were captured using a fluorescence microscope (BZ-X700, KEYENCE; Carl Zeiss, Oberkochen, Germany).

**Western blot**

Embryos were removed from a pregnant ddY mouse (Japan SLC) at 14 days of gestation. Hippocampal neurons were cultured for 3 days, followed by treatments with 1 µM diosgenin or vehicle solution (0.1% ethanol) for 4 days in 35 mm-dishes. The neurons were homogenized with M-PER (Thermo Scientific) containing 1×Halt protease and phosphatase inhibitor cocktail (Thermo Scientific). The neuron lysate (5 µg/lane) was loaded on SDS-PAGE for western blot analysis. After blocking the membrane with 5% skim milk (Wako Pure Chemical Industries, Japan) in 0.1% Tween-TBS for 30 min at room temperature, goat anti-mouse SPARC antibodies (1:1,000, R&D systems, catalog no. AF942) and mouse anti-GAPDH antibodies (1:1,000, Applied biological materials, catalog no. G041) were used as the primary antibodies, and horseradish peroxidase (HRP)-conjugated donkey anti-goat immunoglobulin G (IgG) (1:2,000, Santa Cruz) and HRP-conjugated goat anti-mouse IgG (1:2,000, Santa Cruz) were used as the secondary antibodies. Amersham™ ECL™ Western Blotting Detection Reagents (Sigma-Aldrich) were used for band detection according to the manufacturer’s protocol. The expression levels of SPARC and GAPDH were quantified using CS Analyzer 3.0 (ATTO, Tokyo, Japan).

**siRNA transfection**

siRNAs were transfected into mouse hippocampal neurons according to the manufacturer’s protocol for nucleofection (Lonza, Basel, Switzerland). Mouse hippocampal neurons (2.5 × 10^5^ cells) were mixed with 30 nM siRNA for SPARC (#S74217, Life Technologies) or 30 nM control siRNA (Select Negative Control siRNA #1, Thermo Scientific) and 0.4 μg GFP vector, and electroporated with an Amaxa 4D-Nucleofector (Lonza). For Supplementary Fig. 6, the neurons were fixed 3 days after siRNA transfection. For Fig. 2G, H, the neurons were treated with 1 µM diosgenin or vehicle solution (0.1% ethanol) for 4 days. The appropriate concentration of siRNA and the appropriate duration for knockdown were determined previously.

**AAV9 injection**

Craniotomy was performed for a limited small section on the bilateral (Fig. 3E–H, Q, R) or unilateral (Fig. 3I–P) CA1 in each mouse placed on a stereotaxic device. Custom AAV9 vectors were engineered and purchased from VectorBuilder (Chicago, IL, USA). AAV9-*Syn1*-Cerulean-WPRE (AAV-Control; AAV-Cont-empty; 10^10^ GC/µl in PBS), AAV9-*Syn1*-mSparc-IRES-Cerulean-WPRE (AAV-SPARC; 10^10^ GC/µl in PBS), AAV9-*Syn1*-hM4Di-T2A-Cerulean-WPRE (AAV-Cont-hM4Di; 10^10^ GC/µl in PBS), or AAV9-*Syn1*-mSparc-P2A-hM4Di-T2A-Cerulean (AAV-Cont-hM4Di; 10^10^ GC/µl in PBS) was injected (1 µl/site) at a speed of 0.5 µl/min into the bilateral CA1 regions (-1.9 mm A-P, +1.7 mm M-L, -1.7 mm D-V) of 5XFAD and wild-type mice.

**Behavioral tests**

In a novel object recognition test, each mouse was given two similar objects that were located at a fixed place within a square box in the training session. After an interval time of 1 h, the test session was performed. In the test session, one of the objects used in the training session was replaced with a novel one (different shape and color compared with the familiar one). The number of times the mice made contact with the two objects within 10 min was recorded in each session. In the object location test, each mouse was given two similar objects that were located at a fixed place within a square box in the training session. Spatial markers (polka dots and vertical stripes) were placed on the walls inside the box. After 1 h, one of the identical objects was relocated to a different place in the test session. The number of times each mouse made contact with the objects within 10 min was recorded in each session. The tests were conducted in a dimly illuminated room (80 lux).

To test locomotor activity, the mice were individually habituated to an open-field box for 10 min. Their paths were tracked using a digital camera system. The total distance travelled (cm), turn angle (degrees), and immobility time (s) were analyzed with EthoVision 3.0 (Noldus, Wageningen, The Netherlands).

**DREADDs experiments**

On the same day of AAV injections to the bilateral CA1 as described above, a shallow hole was made in the skull corresponding to the location of the cerebellum, and a micro anchor screw (1.6 mm; Bio Research Center, Nagoya, Japan) was softly placed onto the hole while ensuring that the cerebral parenchyma was not penetrated. Craniotomy was performed overlying the bilateral PFC, and a guide cannula (micro slim A-I, ID: 0.4 mm, OD: 0.5 mm DV: 2.5 mm; Eicom, Kyoto, Japan) was implanted into the center position covering the bilateral PFC (+2.0 mm A-P, +0.0 mm M-L, -2.5 mm D-V). To avoid hemorrhage, the guide cannula was first inserted at a site slightly lateral to the superior sagittal sinus, and it was horizontally moved to +0.0 mm M-L using a stereotaxic device. The guide cannula was cemented, and a dummy cannula (Eicom) was filled into the guide cannula until the behavioral experiments.

Novel object recognition tests were performed on 21 days and 23 days after operations with microinjection of saline or clozapine-N-oxide (CNO) into the PFC. After the training session, mice were briefly anesthetized with isoflurane, and 0.3 µl saline or 0.3 µl 1 mM CNO (in saline) was injected into the PFC using a cannula (Eicom) that was inserted into the guide cannula at a rate of 0.5 µl/min. All the mice were checked to confirm wakefulness, and the test session was performed 1 h after the training session. The number of times the mice made contact with the two objects within 10 min was recorded in each session.

**Anterograde labeling using BDA**

After drug administration to the mice as described above, 10% BDA (in PBS, Thermo Scientific, catalog no. D1956) was injected into the CA1 region (-1.9 mm A-P, +1.7 mm M-L, -1.7 mm D-V) at a speed of 0.5 ml/min in 5XFAD and wild-type mice (female, 6 months old). Seven days after BDA injection, the mice were sacrificed to perform immunohistochemical analysis.

**Immunohistochemistry**

Mice were deeply anesthetized with isoflurane and perfused with 20 ml ice-cold saline. Their brains were carefully removed from the skull and stored at -30°C. The brains were cut into 20-µm coronal slices using a cryostat. The slices were fixed with 4% paraformaldehyde for 90 min and immunostained at 4°C for 24 h in 0.5% Triton X-PBS with the following primary antibodies: mouse anti-GFAP (1:1,000, Sigma, catalog no. G3893), chicken anti-NF-H (1:1,000, Merck Millipore, catalog no. AB5539), mouse anti-NeuN (1:200, Merck Millipore, catalog no. MAB377), goat anti-mouse SPARC (1:100, R&D systems, catalog no. AF942), mouse anti-hypophosphorylated NF-H (pNF-H; 1:250, Covance, catalog no. SMI 35), goat anti-Synaptophysin (1:200, Frontier Institute co., ltd., Japan, catalog no. MSFR105710), rabbit anti-PSD95 (1:200, Frontier Institute co., ltd., catalog no. MSFR106770), and rabbit anti-collagen I (1:500, Abcam, catalog no. ab34710) antibody. Secondary antibodies—Alexa 488-, 568-, 594-, or 647-conjugated affinity-purified secondary anti-mouse IgG, anti-rabbit IgG, anti-goat IgG, and anti-chicken IgY (1:400)—were reacted for 2 h at room temperature in 0.5% Triton X-PBS. For the detection of BDA, the slices were immunostained at room temperature for 2 h with Alexa 594-conjugated Streptavidin (1:1,000, Thermo Scientific, catalog no. S11227) in 0.5% Triton X-PBS. Staining with 1 µg/ml DAPI (in PBS) was performed at room temperature for 10 min. Fluorescence images were captured using a fluorescence microscope (BZ-X700, KEYENCE, Osaka, Japan).

**Collagen type I coating and measurement of axonal length**

To analyze SPARC overexpression-induced axonal growth in collagen I coating, 30 µg/ml Cellmatrix I-C (Wako, Japan) in 10^-3^ M hydrochloric acid (HCl) was further coated on the PDL (5 mg/mL; Sigma-Aldrich, St. Louis, MO, USA)-coated 8-well slides for 1 h (on ice). After washing with Hanks' balanced salt solution twice and with Neurobasal medium (Thermo Scientific) once, the primary cultured hippocampal neurons were seeded on the slides (Fig. 4J). For partial coating of collagen I on the slides, a grid seal (Iwaki, Japan) was pasted on the back side of the PDL-coated 4-well slides, and Cellmatrix I-C (in 10^-3^ M HCl) was partially coated on the right side of the slides (limited on axon-growing regions) by a micropipette with the help of the grids (on ice; Fig. 4K). One hour after collagen I coating, the slide was washed and a Culture-Inserts 3 Well (Ibidi, Germany) was placed on the slides. Next, the primary cultured hippocampal neurons were seeded on the middle separated well. After neurons attached onto the slides, the Culture-Inserts 3 Well was removed, and AAV9 vectors were added to the neurons as described above for 14 days.

**Live cell imaging using triple chamber neuron device**

Cover glasses were sonicated for 30 min in dH_2_O, washed once with ethanol, and three times with dH_2_O. PDL was applied on the cover glasses and incubated in a 10% CO_2_ incubator (37 °C) overnight. The cover glasses were washed five times with dH_2_O and dried. A triple chamber neuron device (Xona Microfluidics, Research Triangle Park, NC, USA) was placed on the cover glass according to the manufacturer’s protocol. Mouse primary hippocampal neurons were seeded on the soma space of a triple chamber neuron device and treated with 5 × 10^7^ GC/µL AAV-control for 10 days. Ten days after culturing, live cell imaging was performed to detect Cerulean-labeled axons in the microgrooves using a fluorescence microscope (BZ-X800, KEYENCE). After that, Aβ_25-35_ (2.5 µM) was treated to soma and axonal spaces for 3 days. Live cell imaging confirmed Cerulean-labeled axons that originally extended into the microgrooves were atrophied by Aβ_25-35_. Then, triple chamber neuron devices were removed from the bottom of the culture dishes, and 5 × 10^6^ GC/µL AAV-control or AAV-SPARC were treated together with 2 µg/mL goat anti-mouse SPARC (R&D systems, catalog no. AF942) or normal goat IgG (control IgG) for 7 days.

**Image analysis**

To quantify the axonal density in the lesion area of axotomized mice brains, a GFAP-positive glial scar was encircled in cortical layers I–IV, and NF-H-positive axonal traces inside the scar were quantified using the image analysis software, ImageJ (Natural Institutes of Health, USA). Axonal length (µm) was divided by the lesion area (µm^2^) for each image. Three to six successive brain slices from the cortex were evaluated per a mouse, and the average value for each mouse was used for analyses. The quantification was performed in a blinded manner.

To investigate axonal growth in the 5XFAD mice brain, the number of Dextran 3000 MW-positive neurons was automatically analyzed using MetaMorph version 7.8 (Molecular Devices, Sunnyvale, CA, USA). The target CA1 and CA3 regions of the dorsal hippocampus (-2.06–-1.82 mm A-P) were encircled, and the number of NeuN-, DAPI-, and tracers-positive cells (Fig. 1, Supplementary Fig. 3–4) or the number of NeuN-positive and tracers-positive cells (Fig. 3I–P, Supplementary Fig. 9) in the region was counted. Three to eight brain slices of the CA1 and CA3 were evaluated per a mouse, and the average value for each mouse was used for analyses.

The expression level of SPARC in NeuN-positive neurons in the CA1 (Stratum oriens, S. pyramidale, S. radiatum, and S. lacunosum moleculare) was automatically quantified using MetaMorph version 7.8 (Fig. 2F, Supplementary Fig. 7, Supplementary Fig. 8F, Supplementary Fig. 10, and Supplementary Fig. 11E).

The length of pNF-H-positive axons in the primary cultured neurons were automatically measured using MetaMorph version 7.8 (Fig. 3D, Fig. 4J, Supplementary Fig. 12A) or an image analyzer Neurocyte (Kurabo, Osaka, Japan) (Fig. 4K). The length of pNF-H-positive axons and expression of SPARC in GFP-positive neurons for siRNA transfection experiment were measured using ImageJ software (Fig. 2H, I, Supplementary Fig. 6). All the axons of GFP-positive neurons were traced by hand and axonal length per neuron was calculated.

The expression level of SPARC in the primary cultured neurons was automatically analyzed using MetaMorph version 7.8. The integrated intensity of SPARC in Map2-positive neuronal cell bodies was quantified for each neuron (Fig. 3B).

The expression of SPARC on the pNF-H-positive axons of primary cultured neurons was analyzed using ImageJ software. All the axons in the image were traced by hand and the expression level of SPARC per axon was calculated (Fig. 4B, Supplementary Fig. 12B, and Supplementary Fig. 14D).

Colocalization of Cerulean-positive axons, synaptophysin-positive pre-synapse, and PSD95-positive post-synapse on NeuN-positive neurons in the PFC were automatically quantified using ImageJ software (Fig. 3H). Synapse density was measured in 30 neurons per a mouse, and the average value for each mouse was included in the final analysis.

BDA-positive axons in brain slices were analyzed using ImageJ software. SPARC expression in the PFC was measured in every BDA-positive axon. The number of SPARC-positive or -negative BDA axons in the PFC was calculated for each mouse.

Densities of axons pursued extracellular collagen I and axons without pursuing extracellular collagen I in Fig. 4M, N were quantified using ImageJ software. All the axons in the original microgroove area and out of the microgroove area in triple neuron device chambers were respectively traced by hand.

**Statistical analysis**

Statistical comparisons were performed using one-way ANOVA with *post-hoc* Bonferroni tests, two-way ANOVA with *post-hoc* Bonferroni test, and unpaired *t*-tests using GraphPad Prism 6 (GraphPad Software, La Jolla, CA, USA). Values of p < 0.05 were considered significant. Data are presented as the mean ± standard error or ± standard deviation of the mean. The sample size was determined to ensure adequate power by using power analysis. The sample size, effect size (*r*), and power (1 - *β*) were written in each Figure legend.

**Supplementary Figure 1. Diosgenin administration promotes axonal growth in cortex-axotomized mice brain.**

Diosgenin (0.1 µmol/kg/day) or vehicle solution was orally administered to cortex-axotomized ddY mice for 15 days. Immunohistochemistry detected NF-H-positive axons (red) and GFAP-positive glial scar (green) in each group (**A**). Axonal density (µm/100 µm^2^ lesion area) (**B**) and lesion area (**C**) were quantified. ***p* < 0.01, two-tailed unpaired *t*-test, mean ± standard deviation, vehicle (Veh), n = 3; diosgenin (Dios), n = 5. (**B**) Effect size (*r*) = 0.864, power (1 - *β*) = 0.979, (**C**) *r* = 0.535, 1 - *β* = 0.327.

NF-H, neurofilament-H.

**Supplementary Figure 2. Two retrograde tracers Dextran 3000 MW injected into the PFC showed similar spread to the HPC in adult ddY mice.**

Twenty-one days after Dextran Texas Red (first tracer) was injected into the PFC of adult ddY mice (female, 8 weeks old), Dextran FITC (second tracer) was further injected into the same region of the PFC.

(**A**) The injection site of the two tracers in the PFC is shown at 7 days after the second tracer injection. The mice in which the injection sites of the two tracers completely matched were used for the experiments. (**B**) The tracers spread into the same neurons in the HPC, confirming the accuracy of the injecting techniques.

PFC, prefrontal cortex; HPC, hippocampus; FITC, fluorescein isothiocyanate.

**Supplementary Figure 3. Expanded data in the hippocampal CA1 region from Figure 1B.**

Seven days after Dextran Texas Red (1^st^ tracing) was injected into the PFC, diosgenin or vehicle solution was orally administered to wild-type and 5XFAD mice once a day for 14 days. Dextran FITC (2^nd^ tracing) was further injected into the PFC at 7 days before sacrifice. High magnification merged images for Dextran Texas Red, Dextran FITC, and NeuN staining in the CA1 were shown. The total number of naïve (Texas Red^+^, FITC^+^, NeuN^+^, DAPI^+^) (**A**), originally projected (Texas Red^+^, NeuN^+^, DAPI^+^) (**B**), growing and naïve (FITC^+^, NeuN^+^, DAPI^+^) (**C**), and NeuN^+^ neurons (**D**) and DAPI^+^ cells (**E**) were quantified. ***p* < 0.01, ****p* < 0.001, *****p* < 0.0001, one-way ANOVA *post-hoc* Bonferroni test, mean ± standard deviation, wild-type mice (Wild)/vehicle (Veh), n = 8; 5XFAD mice (5XFAD)/Veh, n = 7; 5XFAD/diosgenin (Dios), n = 8. (**A**) Effect size (*r*) = 0.912, power (1 - *β*) = 0.961, (**B**) *r* = 0.765, 1 - *β* = 0.869, (**C**) *r* = 0.901, 1 - *β* = 0.956, (**D**) *r* = 0.168, 1 - *β* = 0.095, (**E**) *r* = 0.526, 1 - *β* = 0.543.

PFC, prefrontal cortex; FITC, fluorescein isothiocyanate; NeuN, neuronal nuclei; DAPI, 4’,6-diamidino-2-phenylindole.

**Supplementary Figure 4. Expanded data in the hippocampal CA3 in Figure 1C.**

Seven days after Dextran Texas Red (1^st^ tracing) was injected into the PFC, diosgenin or vehicle solution was orally administered to wild-type and 5XFAD mice once a day for 14 days. Dextran FITC (2^nd^ tracing) was further injected into the PFC at 7 days before sacrifice. High magnification merged images for Dextran Texas Red, Dextran FITC, and NeuN staining in the CA3 were shown. The total number of naïve (Texas Red^+^, FITC^+^, NeuN^+^, DAPI^+^) (**A**), originally projected (Texas Red^+^, NeuN^+^, DAPI^+^) (**B**), growing and naïve (FITC^+^, NeuN^+^, DAPI^+^) (**C**), and NeuN^+^ neurons (**D**) and DAPI^+^ cells (**E**) were quantified. ****p* < 0.001, *****p* < 0.0001, one-way ANOVA *post-hoc* Bonferroni test, mean ± standard deviation, wild-type mice (Wild)/vehicle (Veh), n = 8; 5XFAD mice (5XFAD)/Veh, n = 7; 5XFAD/diosgenin (Dios), n = 8. (**A**) Effect size (*r*) = 0.890, power (1 - *β*) = 0.952, (**B**) *r* = 0.817, 1 - *β* = 0.911, (**C**) *r* = 0.825, 1 - *β* = 0.917, (**D**) *r* = 0.426, 1 - *β* = 0.377, (**E**) *r* = 0.431, 1 - *β* = 0.385.

PFC, prefrontal cortex; FITC, fluorescein isothiocyanate; NeuN, neuronal nuclei; DAPI, 4’,6-diamidino-2-phenylindole.

**Supplementary Figure 5. Full gel image of Figure 2D.**

Mouse primary hippocampal neurons were cultured for 3 days and treated with diosgenin (1 µM) or vehicle solution for 4 days. The neuron lysates were used for western blot analysis. The estimated molecular weight of SPARC is approximately 43 k.

SPARC, secreted protein acidic and rich in cysteine.

**Supplementary Figure 6. Knockdown of SPARC was induced by siRNA transfection in neurons.**

siRNA for SPARC (30 nM; siSPARC) or control siRNA (30 nM; siControl) was transfected together with GFP vector into mouse primary hippocampal neurons for 3 days. (**B**) The expression level of SPARC and (**C**) pNF-H-positive axonal length in GFP^+^ neurons (green arrowheads) were quantified in each group. ****p* < 0.001, two-tailed unpaired *t*-test, mean ± standard error. (**B**) n = 40–59 neurons, Effect size (*r*) = 0.380, power (1 - *β*) = 0.982. (**C**) n = 17–24 photos, *r* = 0.123, 1 - *β* = 0.051.

**Supplementary Figure 7. SPARC was overexpressed in the hippocampal neurons by injection of AAV-SPARC in mice.**

AAV-SPARC or AAV-Control was injected into the hippocampal CA1 of wild-type mice. The expression level of SPARC in CA1 neurons was assessed at 14 (**A**), 21 (**B**), and 28 (**C**) days after AAV injection using immunocytochemistry. AAV-derived Cerulean was detected at the injection sites, and expression of SPARC in NeuN-positive neurons was quantified in the hippocampal CA1 region in each group. ****p* < 0.001, two-tailed unpaired *t*-test, mean ± standard deviation, n = 6/group. (**A**) Effect size (*r*) = 0.831, power (1 - *β*) = 0.996, (**B**) *r* = 0.885, 1 - *β* = 0.974, (**C**) *r* = 0.849, 1 - *β* = 0.999.

SPARC, secreted protein acidic and rich in cysteine; AAV, adeno-associated virus; NeuN, neuronal nuclei.

**Supplementary Figure 8. Locomotor activity and body weight were not changed by AAV-SPARC injection in 5XFAD mice.**

Wild-type and 5XFAD mice were injected with 10^10^ GC of AAV-Control or AAV-SPARC in the hippocampal CA1 region. A locomotion test was performed at 24 days after AAV injection. The total distance travelled (cm) (**A**), turn angle (degrees) (**B**), and immobility time (s) (**C**) did not differ among the groups. *p* > 0.05, one-way ANOVA *post-hoc* Dunnett’s test. (**A**) Effect size (*r*) = 0.468, power (1 - *β*) = 0.343, (**B**) *r* = 0.269, 1 - *β* = 0.139, (**C**) *r* = 0.531, 1 - *β* = 0.429. (**D**) The body weight (g) of mice was not changed by the AAV injections. *p* > 0.05, AAV injection × day interaction, repeated-measures two-way ANOVA *post-hoc* Bonferroni test, mean ± standard deviation, n = 6 mice/group.

**E, F**, After the behavioral tests, expression of SPARC in NeuN-positive neurons was quantified in the hippocampal CA1 region in each group. ***p* < 0.01, ****p* < 0.001, one-way ANOVA *post-hoc* Bonferroni test, mean ± standard deviation, n = 6 mice/group. (**F**) *r* = 0.821, 1 - *β* = 0.809.

**G**, After the behavioral tests, AAV-derived Cerulean-positive axonal shafts and terminals of the hippocampal CA1 neurons were observed in the PFC region in each group. Yellow arrowheads indicate fiber-like axons.

SPARC, secreted protein acidic and rich in cysteine; AAV, adeno-associated virus.

**Supplementary Figure 9. SPARC overexpression in neurons promoted axonal growth from the CA3 to the PFC in 5XFAD mouse brains.**

Seven days after Dextran Texas Red (1^st^ tracing) was injected into the PFC of wild-type and 5XFAD mice, 10^10^ GC of AAV-Control or AAV-SPARC was injected into the hippocampal CA1 region. After 21 days, Dextran FITC (2^nd^ tracing) was further injected into the PFC. Seven days after the 2^nd^ tracer injection, the number of axon-growing (Dextran^-^, FITC^+^, NeuN^+^) neurons (**A**), axon-degenerating (Dextran^+^, FITC^-^, NeuN^+^) neurons (**B**), naïve (Texas Red^+^, FITC^+^, NeuN^+^) neurons (**C**), originally projected (Texas Red^+^, NeuN^+^) neurons (**D**), regenerated and naïve (FITC^+^, NeuN^+^) neurons (**E**), NeuN^+^ neurons (**F**) in the hippocampal CA3 region were quantified. ****p* < 0.001, *****p* < 0.0001, one-way ANOVA *post-hoc* Bonferroni test, mean ± standard deviation, n = 6 mice/group. (**A**) Effect size (*r*) = 0.934, power (1 - *β*) = 0.921, (**B**) *r* = 0.860, 1 - *β* = 0.871, (**C**) *r* = 0.916, 1 - *β* = 0.910, (**D**) *r* = 0.893, 1 - *β* = 0.895, (**E**) *r* = 0.868, 1 - *β* = 0.877, (**F**) *r* = 0.310, 1 - *β* = 0.180.

PFC, prefrontal cortex; FITC, fluorescein isothiocyanate; SPARC, secreted protein acidic and rich in cysteine.

**Supplementary Figure 10. SPARC was overexpressed in the hippocampal neurons after the injection of AAV-SPARC in mice.**

AAV-SPARC-hM4Di or AAV-Control-hM4Di was injected into the hippocampal CA1 of wild-type mice. The expression level of SPARC in CA1 neurons was assessed at 21 (**A**) and 28 (**B**) days after AAV injection using immunocytochemistry. AAV-derived Cerulean was detected at the injection sites, and expression of SPARC in NeuN-positive neurons was quantified in the hippocampal CA1 region in each group. ****p* < 0.001, two-tailed unpaired *t*-test, mean ± standard deviation, n = 6/group. (**A**) Effect size (*r*) = 0.683, power (1 - *β*) = 0.830, (**B**) *r* = 0.816, 1 - *β* = 0.992. AAV9-derived Cerulean-positive axons (magenta) and NeuN-positive neurons (green) were confirmed in the PFC region.

SPARC, secreted protein acidic and rich in cysteine; AAV, adeno-associated virus; NeuN, neuronal nuclei.

**Supplementary Figure 11. Locomotor activity and body weight were not changed by AAV-SPARC injection in 5XFAD mice.**

Wild-type and 5XFAD mice were inserted with 10^10^ GC of AAV-Cont-empty, AAV-Cont-hM4Di, or AAV-SPARC-hM4Di in the hippocampal CA1. At the same time, a cannula was infused into the center position covering the right and left PFC. A locomotion test was performed at 24 days after AAV injection. The total distance travelled (cm) (**A**), turn angle (degrees) (**B**), and immobility time (s) (**C**) did not differ among the groups. *p* > 0.05, one-way ANOVA *post-hoc* Dunnett’s test, mean ± standard deviation, n = 3–5 mice/group. (**A**) Effect size (*r*) = 0.488, power (1 - *β*) = 0.298, (**B**) *r* = 0.597, 1 - *β* = 0.431, (**C**) *r* = 0.463, 1 - *β* = 0.270. (**D**) After the behavior tests, infused cannula in the PFC was observed in light field images. (**E, F**) Expression of SPARC in NeuN-positive neurons was quantified in the hippocampal CA1 region in each group. ***p* < 0.01, ****p* < 0.001, *****p* < 0.0001, one-way ANOVA *post-hoc* Bonferroni test, mean ± standard deviation, n = 3–5 mice/group. (**F**) *r* = 0.903, 1 - *β* = 0.807. AAV9-derived Cerulean-positive axons (magenta) and NeuN-positive neurons (green) were confirmed in the PFC region.

SPARC, secreted protein acidic and rich in cysteine; AAV, adeno-associated virus; NeuN, neuronal nuclei.

**Supplementary Figure 12. Diosgenin-induced axonal regrowth and SPARC upregulation on axons were mediated by 1,25D_3_-MARRS.**

Mouse primary hippocampal neurons were cultured for 3 days and then treated with or without Aβ_25-35_ (2.5 µM) for 3 days. Next, neurons were treated with diosgenin (Dios; 1 µM) or vehicle solution (Veh) together with 1,25D_3_-MARRS neutralizing antibody (MARRS Ab) or control IgG (IgG) for 4 days. (**A**) pNF-H-positive axonal length and (**B**) the expression level of SPARC on axons were quantified in each group. ***p* < 0.01, *****p* < 0.0001, one-way ANOVA *post-hoc* Bonferroni test, mean ± standard error. (**A**) n = 13–18 photos, Effect size (*r*) = 0.756, power (1 - *β*) = 0.999, (**B**) n = 57–136 neurons, *r* = 0.568, 1 - *β* = 1.

1,25D_3_-MARRS, 1,25D_3_-membrane-associated rapid response steroid-binding receptor; pNF-H, hypophosphorylated neurofilament-H.

**Supplementary Figure 13. Anterograde labeling with BDA detected axons projected from the HPC to the PFC.**

Anterograde tracer BDA was injected into the hippocampal CA1 region. After 7 days, the brain slices were immunostained with Alexa 594-conjugated Streptavidin. (**A**) The injection site of BDA and DAPI in the CA1 are shown. (**B**) BDA were immunostained with pNF-H-positive axons in the PFC. Almost all BDA (red) were co-localized with axons (green) in the PFC (white arrowheads).

HPC, hippocampus; PFC, prefrontal cortex; BDA, biotinylated dextran amines; DAPI, 4’,6-diamidino-2-phenylindole; pNF-H, hypophosphorylated neurofilament-H.

**Supplementary Figure 14. Long-term-cultured mature neurons expressed collagen type I near axons. Aβ_35-25_ did not influence on colocalization of axons with collagen I and expression of SPARC on axons.**

**A, B**, Mouse primary hippocampal neurons were cultured for 10 days (immature) (**A**) or 21 days (mature) (**B**), and immunostained with pNF-H (green) and collagen type I (collagen I) (red), respectively. While immature neurons hardly expressed collagen I, mature neurons showed elevated levels of collagen I that co-localized with pNF-H-positive axons.

**C**–**E**, Mouse primary hippocampal neurons were cultured for 14 days and treated with Aβ_25-35_ or a negative control peptide for Aβ_25-35_, Aβ_35-25_ (2.5 µM) for 3 days. Then, neurons were cultured in normal medium for 4 days. (**C**) SPARC signal was not detected when 2^nd^ antibody alone for anti-SPARC antibody (2^nd^ anti-goat IgG) was used for immunocytochemistry. (**D**) Length of axons colocalized with collagen I (pNF-H^+^, collagen I^+^) and (**E**) expression of SPARC on axons were measured in each group. *****p* < 0.0001, one-way ANOVA *post-hoc* Bonferroni test, mean ± standard error. (**D**) n = 12 images/group, Effect size (*r*) = 0.995, power (1 - *β*) = 0.999, (**E**) n = 73–132 axons/group, *r* = 0.368, 1 - *β* = 0.999.

**F**, Mouse primary hippocampal neurons were cultured for 3 days and then treated with or without Aβ_25-35_ (2.5 µM) for 3 days. Next, neurons were treated with diosgenin (0.1 or 1 µM) or vehicle solution for 4 days. The number of neurons was measured for each treatment. ****p* < 0.001, *****p* < 0.0001 vs Aβ_25-35_ (Aβ)/Vehicle (Veh), one-way ANOVA *post-hoc* Dunnett’s test, mean ± standard error, n = 12–16 images/group, *r* = 0.678, 1 - *β* = 0.991.

pNF-H, hypophosphorylated neurofilament-H.

**Supplementary Table 1. Genes differentially expressed in axon-growing neurons compared with naïve neurons in 5XFAD mice brains.**

**Supplementary Table 2. All gene expression profiles in axon-growing neurons compared with naïve neurons in 5XFAD mice brain.**
