# Supplementary material for: Diosgenin restores memory function via SPARC-driven axonal growth from the hippocampus to the PFC in Alzheimer’s disease model mice
Source: Mol Psychiatry. 2023 Apr 22;28(6):2398–411. doi: 10.1038/s41380-023-02052-9 (PMC10611574; doi:10.1038/s41380-023-02052-9)
Supplement: Supplementary file 2 — Supplementary Table 1 [file 41380_2023_2052_MOESM2_ESM.docx]

| **Supplemental Table 1.** **Genes differentially expressed in axon-growing neurons compared with naïve neurons in 5XFAD mice brains.** | | | | | |
| --- | --- | --- | --- | --- | --- |
| **NO.** | **Axon-growing**  **Avg (log2)** | **Naïve**  **Avg (log2)** | **Fold Change** | **Gene Symbol** | **Description** |
| 1 | 15.44 | 11.44 | **15.95** | Sparc | secreted acidic cysteine rich glycoprotein |
| 2 | 14.4 | 10.54 | **14.53** | Klk6 | kallikrein related-peptidase 6 |
| 3 | 11.85 | 8.08 | **13.64** | Mia | melanoma inhibitory activity |
| 4 | 12.98 | 9.6 | **10.38** | Ermard | ER membrane associated RNA degradation |
| 5 | 11.34 | 8.06 | **9.73** | Psmd10 | proteasome (prosome, macropain) 26S subunit, non-ATPase, 10 |
| 6 | 12.13 | 9.01 | **8.71** | Tgm1 | transglutaminase 1, K polypeptide |
| 7 | 11.05 | 8.03 | **8.12** | Spp1 | secreted phosphoprotein 1 |
| 8 | 13.05 | 10.12 | **7.6** | Arpc1b | actin related protein 2/3 complex, subunit 1B |
| 9 | 12.71 | 9.8 | **7.49** | Gm3435 | predicted gene 3435 |
| 10 | 13.7 | 10.8 | **7.47** | Cxcl16 | chemokine (C-X-C motif) ligand 16 |
| 11 | 11.95 | 9.08 | **7.32** | Sv2a | synaptic vesicle glycoprotein 2 a |
| 12 | 11.78 | 8.93 | **7.23** | Fam183b | family with sequence similarity 183, member B |
| 13 | 14.9 | 12.14 | **6.79** | Lgals1 | lectin, galactose binding, soluble 1 |
| 14 | 11.37 | 8.61 | **6.77** | Bst2 | bone marrow stromal cell antigen 2 |
| 15 | 11.9 | 9.14 | **6.77** | Stxbp6 | syntaxin binding protein 6 (amisyn) |
| 16 | 14.77 | 12.05 | **6.59** | 9030025P20Rik | RIKEN cDNA 9030025P20 gene |
| 17 | 13.15 | 10.45 | **6.52** | Pmp22 | peripheral myelin protein 22 |
| 18 | 11.1 | 8.46 | **6.23** | Mrpl27 | mitochondrial ribosomal protein L27 |
| 19 | 10.67 | 8.05 | **6.14** | Mlh1 | mutL homolog 1 (E. coli) |
| 20 | 11.26 | 8.66 | **6.08** | Med7 | mediator complex subunit 7 |
| 21 | 11.68 | 9.11 | **5.91** | Sdhb | succinate dehydrogenase complex, subunit B, iron sulfur (Ip) |
| 22 | 11.53 | 8.97 | **5.9** | Ntng1 | netrin G1 |
| 23 | 10.6 | 8.08 | **5.76** | Sycp1 | synaptonemal complex protein 1 |
| 24 | 12.36 | 9.84 | **5.73** | Polr2e | polymerase (RNA) II (DNA directed) polypeptide E |
| 25 | 12.26 | 9.74 | **5.71** | Ccnc | cyclin C |
| 26 | 11.13 | 8.65 | **5.57** | Plekha1 | pleckstrin homology domain containing, family A (phosphoinositide binding specific) member 1 |
| 27 | 12.46 | 10.02 | **5.42** | Susd4 | sushi domain containing 4 |
| 28 | 11.28 | 8.86 | **5.37** | Zfand2a | zinc finger, AN1-type domain 2A |
| 29 | 13.36 | 10.94 | **5.36** | 0610009B22Rik | RIKEN cDNA 0610009B22 gene |
| 30 | 10.94 | 8.54 | **5.28** | Rab33b | RAB33B, member RAS oncogene family |
| 31 | 12.21 | 9.83 | **5.21** | Psma1 | proteasome (prosome, macropain) subunit, alpha type 1 |
| 32 | 11.09 | 8.72 | **5.17** | Pbk | PDZ binding kinase |
| 33 | 13.08 | 10.72 | **5.14** | Fcgr3 | Fc receptor, IgG, low affinity III |
| 34 | 10.8 | 8.44 | **5.12** | Nipsnap3b | nipsnap homolog 3B (C. elegans) |
| 35 | 10.96 | 8.61 | **5.11** | Gng8 | guanine nucleotide binding protein (G protein), gamma 8 |
| 36 | 10.39 | 8.03 | **5.11** | Nudt22 | nudix (nucleoside diphosphate linked moiety X)-type motif 22 |
| 37 | 11.77 | 9.42 | **5.09** | Pycard | PYD and CARD domain containing |
| 38 | 10.52 | 8.18 | **5.05** | Stk19 | serine/threonine kinase 19 |
| 39 | 15.33 | 13 | **5.04** | Cd9 | CD9 antigen |
| 40 | 11.57 | 9.24 | **5.01** | D11Wsu47e | DNA segment, Chr 11, Wayne State University 47, expressed |
| 41 | 11.32 | 13.69 | **-5.18** | Ppp1r9b | protein phosphatase 1, regulatory subunit 9B |
| 42 | 9.14 | 11.52 | **-5.2** | Zhx1 | zinc fingers and homeoboxes 1 |
| 43 | 10.32 | 12.7 | **-5.22** | Ccdc88c | coiled-coil domain containing 88C |
| 44 | 8.52 | 10.91 | **-5.24** | Fmo1 | flavin containing monooxygenase 1 |
| 45 | 10.74 | 13.16 | **-5.35** | Bag5 | BCL2-associated athanogene 5 |
| 46 | 8.13 | 10.57 | **-5.43** | Zdhhc2 | zinc finger, DHHC domain containing 2 |
| 47 | 9.49 | 11.95 | **-5.53** | Crip1 | cysteine-rich protein 1 (intestinal) |
| 48 | 10.19 | 12.71 | **-5.71** | Wfs1 | Wolfram syndrome 1 homolog (human) |
| 49 | 9.13 | 11.7 | **-5.92** | Gmcl1 | germ cell-less homolog 1 (Drosophila) |
| 50 | 9.39 | 11.99 | **-6.06** | Mpp6 | membrane protein, palmitoylated 6 (MAGUK p55 subfamily member 6) |
| 51 | 8.38 | 10.99 | **-6.09** | Uhmk1 | U2AF homology motif (UHM) kinase 1 |
| 52 | 8.65 | 11.3 | **-6.28** | Flna | filamin, alpha |
| 53 | 9.01 | 11.67 | **-6.31** | Sgms1 | sphingomyelin synthase 1 |
| 54 | 9.29 | 12 | **-6.58** | Zfp609 | zinc finger protein 609 |
| 55 | 8.99 | 11.71 | **-6.62** | Alkbh8 | alkB, alkylation repair homolog 8 (E. coli) |
| 56 | 9.74 | 12.5 | **-6.74** | Mpped1 | metallophosphoesterase domain containing 1 |
| 57 | 8.72 | 11.56 | **-7.17** | Fam60a | family with sequence similarity 60, member A |
| 58 | 9.81 | 12.68 | **-7.32** | Pwwp2a | PWWP domain containing 2A |
| 59 | 8.15 | 11.13 | **-7.88** | B630005N14Rik | RIKEN cDNA B630005N14 gene |
| 60 | 8.34 | 11.45 | **-8.65** | Nbr1 | neighbor of Brca1 gene 1 |
| 61 | 8.72 | 11.87 | **-8.87** | Actr3b | ARP3 actin-related protein 3B |
| 62 | 8.38 | 11.57 | **-9.09** | Neurod6 | neurogenic differentiation 6 |
| 63 | 10.17 | 13.36 | **-9.1** | Ndnf | neuron-derived neurotrophic factor |
| 64 | 8.5 | 11.85 | **-10.21** | Pcmt1; BC020402 | protein-L-isoaspartate (D-aspartate) O-methyltransferase 1; cDNA sequence BC020402 |
| 65 | 8.28 | 11.98 | **-13.06** | Mapk10 | mitogen-activated protein kinase 10 |
| 66 | 9.06 | 13.1 | **-16.43** | Sfxn5 | sideroflexin 5 |
| 67 | 11.56 | 16.22 | **-25.26** | Gm11096 | predicted gene 11096 [Source:MGI Symbol;Acc:MGI:3779332] |
